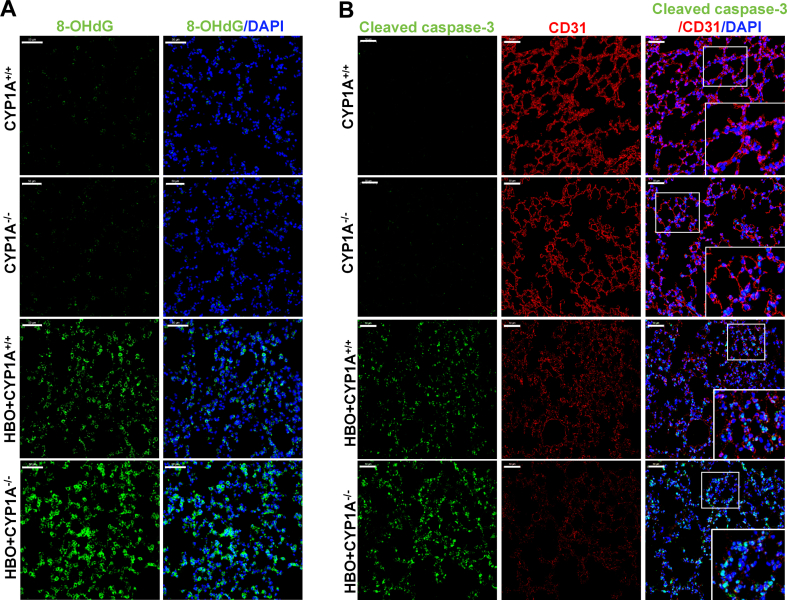# The CYP1A-HSF1 axis alleviates mitochondrial oxidative stress to limit HBO-induced lung endothelial barrier damage

**DOI:** 10.1016/j.redox.2026.104308

**Published:** 2026-07-16

**Authors:** Jing-Qian Huang, Peng-Xiang Wang, Xiao-Yong Zhang, Yi-Ting You, Jun-Hui Zhan, Dan-Hong Xu, Yu-Jian Liu, Xiao-Yan Zhu, Xiao-Chen Bao

**Affiliations:** a Department of Physiology, Naval Medical University, Shanghai, 200433, China; b School of Kinesiology, The Key Laboratory of Exercise and Health Sciences of Ministry of Education, Shanghai University of Sport, Shanghai, 200438, China; c Department of Anesthesiology and Surgical Intensive Care Unit, Xinhua Hospital, Shanghai Jiao Tong University School of Medicine, Shanghai, 200092, China; d Department of Diving Medicine, Naval Medical Center, Shanghai, 200082, China

**Keywords:** Hyperbaric oxygen, CYP1A, Lung injury, HSF1, Mitochondrial dysfunction

## Abstract

Although hyperbaric oxygen (HBO) therapy is an established medical intervention effective for various conditions, prolonged or excessive exposure can induce acute lung injury (ALI), the mechanisms of which remain poorly understood. In this study, we show that expression of CYP1A1 and CYP1A2, two isoforms of the cytochrome P450 (CYP) 1A subfamily, is significantly upregulated in mouse lung tissues and primary mouse lung microvascular endothelial cells (MLVECs) following HBO exposure. Global double knockout of CYP1A1 and CYP1A2 (referred to as CYP1A-deficient mice) exacerbated pulmonary endothelial dysfunction and ALI in response to HBO, whereas CYP1A inducer β-naphthoflavone (BNF) ameliorated these pathological changes. Mechanistically, activation of the heat shock factor 1 (HSF1) signaling pathway mediates the protective effects of CYP1A induction against HBO-induced mitochondrial oxidative stress, mitochondrial dysfunction, and endothelial cell apoptosis. Critically, HSF1 inhibition abolished BNF-induced HSF1 recruitment to the succinate dehydrogenase subunit C (SDHC) promoter, thereby suppressing SDHC transcription in HBO-exposed pulmonary endothelial cells. Furthermore, the HSF1-mediated protection is dependent on succinate dehydrogenase C (SDHC), thereby linking CYP1A upregulation to the preservation of pulmonary endothelial barrier integrity. CYP1A deficiency leads to reduced SDHC expression, resulting in enhanced mitochondrial oxidative stress, impaired mitochondrial function, and increased apoptosis in lung tissues under HBO conditions. Our findings reveal a previously unrecognized protective mechanism in which upregulation of CYP1A serves as an adaptive response against HBO-induced pulmonary endothelial injury by attenuating mitochondrial oxidative stress and restoring mitochondrial homeostasis via the HSF1/SDHC signaling axis.

## Highlights

• CYP1A upregulation serves as a protective response against HBO-induced ALI.
• HSF1 mediates the protective effects of CYP1A induction against HBO-induced ALI.
• The CYP1A-mediated protection is dependent on HSF1/SDHC signaling pathway.
• CYP1A/HSF1/SDHC axis mitigates mitochondrial dysfunction and endothelial apoptosis.
• Targeting the CYP1A/HSF1/SDHC pathway offers a strategy against HBO-induced ALI.

## Graphical abstract

**Figure:**
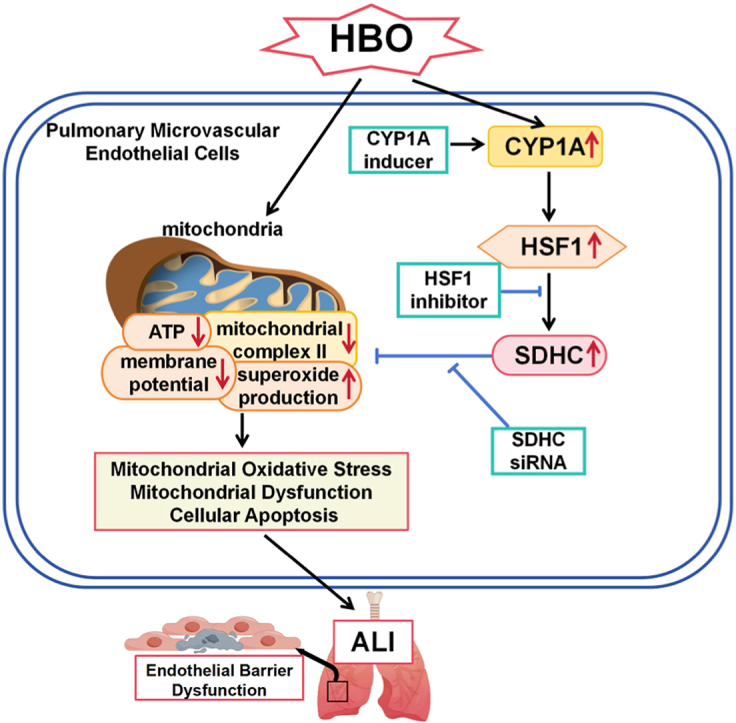

## 1 Introduction

Hyperbaric oxygen (HBO) involves breathing pure oxygen at pressures greater than 1 atm. The Undersea and Hyperbaric Medical Society (UHMS) recognizes HBO therapy for conditions like decompression sickness, carbon monoxide poisoning, diabetic wounds, and delayed radiation injury. However, excessive oxygen inhalation disrupts the body's oxidation-antioxidant balance, leading to oxidative stress and damage [1]. Long-term exposure to high oxygen concentrations can cause oxygen toxicity, resulting in seizures, pulmonary insufficiency, and retinopathy of prematurity [2]. At 2 ATA or lower, HBO primarily damages the lungs [3]. Studies show that mice exposed to HBO exhibit increased inflammation, apoptosis, epithelial injury, and endothelial dysfunction, causing acute lung injury (ALI) [4]. Critically, the molecular pathogenesis of HBO-induced ALI differs fundamentally from that of sepsis- or virus-induced ALI. In sepsis-induced ALI, pathogen-associated molecular patterns (PAMPs) activate pattern recognition receptors (PRRs), such as Toll-like receptors (TLRs), triggering a cytokine storm [5]. In viral pneumonia-induced ALI, direct viral invasion of alveolar epithelial cells and macrophages drives immune evasion, unchecked viral replication, and a massive cytokine storm [6]. In contrast, HBO-induced ALI is initiated by direct oxidative stress that overwhelms intracellular antioxidant systems, generating massive reactive oxygen species (ROS) and lipid peroxidation products before significant inflammatory cell recruitment [2,[7], [8], [9], [10]]. However, the exact mechanisms of HBO-induced ALI remain unclear, and effective prevention and treatment strategies are lacking. Further research into its pathogenesis and management is urgently needed.

The cytochrome P450 (CYP) enzyme superfamily is a group of membrane-bound heme proteins with monooxygenase activity. These enzymes are the primary metabolic enzymes in lung tissue, responsible for the metabolism of both exogenous substances (e.g., drugs and environmental pollutants) and endogenous substances (e.g., steroids and fatty acids) [11]. In recent years, studies on ALI models have predominantly focused on the CYP1 family, a branch of the CYP superfamily that includes the CYP1A and CYP1B1 subfamilies. The CYP1A subfamily further comprises two isoforms, CYP1A1 and CYP1A2 [[12], [13], [14]]. However, research findings regarding the role of the CYP1 family in oxygen toxicity pathogenesis remain contradictory. Studies using combined CYP1A1/CYP1A2 gene knockout mice and experiments involving CYP1A agonists and antagonists have demonstrated that CYP1A protects against hyperoxia-induced lung injury [[15], [16], [17], [18], [19]]. Conversely, CYP1B1 activation has been identified as a key pathogenic mechanism in hyperoxic lung injury [20] These results underscore the distinct roles of CYP1 subfamily members in oxygen toxicity. However, whether CYP1 family enzymes contribute to the pathogenesis of HBO-induced ALI remains unclear and warrants further investigation.

In this study, we first demonstrated that CYP1A1 and CYP1A2, but not CYP1B1, are upregulated in pulmonary vascular endothelial cells and murine lung tissues exposed to HBO. We subsequently investigated the effects of double deficiency of CYP1A1 and CYP1A2, as well as the impact of a CYP1A inducer, on HBO-induced lung endothelial barrier dysfunction. Furthermore, we elucidated the molecular mechanisms underlying the protective effects of CYP1A on endothelial cells in the context of HBO-induced injury. Our findings reveal a novel and critical role of the CYP1A-HSF1 axis in mitigating HBO-induced mitochondrial oxidative stress and highlight potential therapeutic targets for HBO-induced ALI.

## 2 Materials and methods

### 2.1 Animals

All experimental mice used in this study were bred and maintained at the Naval Medical Research Center. Prior to the experiments, the mice were housed in individually ventilated cages and acclimatized to the facility for one week. All experimental procedures were conducted in accordance with the Animal Welfare Ethical Review Guidelines and were approved by the Institutional Animal Care and Use Committee (Ethics number: NMC-2021021). Global CYP1A1 and CYP1A2 double gene knockout (CYP1A^−/−^) mice were obtained from Shanghai Southern Model Organisms (Shanghai, China), generated using CRISPR/Cas9 technology. The gene mutations were introduced via non-homologous end-joining repair, resulting in functional inactivation of both CYP1A1 and CYP1A2. Deletion of these genes was confirmed by PCR analysis of genomic DNA using the following primer pairs: (5′-ATCCCCTCAGGTCCCTATTTTCAC-3′) and (5′-GGCCGTCACCCTTGTTTCTTTT-3′) for CYP1A1, and (5′-TCCTCAGGTCCCTATTTTCAC-3′) and (5′-GGCAGCTCCCACTTGTCA-3′) for CYP1A2. Notably, randomization was performed using a random number table method during animal experimentation. Throughout the in vivo measurement and analysis phases, both genotype and treatment information were blinded to minimize experimental bias. To eliminate potential confounding effects due to sex differences, only male mice were included in the experiments.

### 2.2 Acute lung injury model induced by hyperbaric oxygen in mice

Hyperbaric oxygen (HBO) treatment was administered as described previously [21]. C57BL/6J mice (8–10 weeks old) were placed inside a hyperbaric chamber (Yantai Hongyuan Oxygen Industry Co., Ltd., China). The chamber was first purged for 5 min with 95 ± 5% O_2_ at a flow rate of 10 L min^−1^. It was then pressurized to an absolute pressure of 0.20 MPa (approximately 2 atm absolute, ATA) at a rate of 0.05 MPa min^−1^. The 6-h HBO exposure period was initiated once the target pressure of 2 ATA was reached. Following the 6-h exposure, decompression to atmospheric pressure was carried out linearly over 20 min. Mice were subsequently removed from the chamber and returned to room air. Lung tissues were collected immediately after HBO exposure for qPCR analysis. Additional lung tissue samples were obtained 24 h after the conclusion of HBO treatment for further analyses.

### 2.3 Isolation and culture of primary mouse pulmonary vascular endothelial cells

MLVECs were isolated from the lungs of 3-week-old male mice. Following previously established protocols [22,23], the mice were anesthetized with 1% sodium pentobarbital (80 mg/kg), and the right ventricle was perfused with cold PBS to remove blood from the pulmonary vasculature. Lung tissue samples, including both peripheral and subpleural regions, were dissected into small tissue blocks of approximately 1 mm^3^, which were evenly distributed onto culture dishes. The culture dishes were then inverted and placed in the incubator for approximately 30 min to allow tissue adherence. Dulbecco's Modified Eagle's Medium (DMEM, Gibco, New York, USA) supplemented with 20% fetal bovine serum (FBS, Gibco, USA) and 1% penicillin-streptomycin-amphotericin B solution (Biosharp, China) was used as the primary culture medium. After 60 h, the tissue blocks were gently removed, and the cells were maintained in basal culture medium supplemented with 10% FBS for routine expansion. MLVECs at passages 2–3 were used for subsequent experimental analyses.

### 2.4 Establishment of an in vitro cell model of HBO-induced acute endothelial cell injury

Endothelial cells were seeded in 6/12/24/48/96-well plates and placed in a hyperbaric chamber (Yantai Hongyuan Oxygen Industry Co., Ltd., China) pre-equilibrated to 37°C. The chamber was purged with 97.5% O_2_/2.5% CO_2_ at a flow rate of 10 L min^−1^ for 5 min, followed by compression to an absolute pressure of 0.20 MPa (≈2 ATA) at a rate of 0.05 MPa min^−1^. The 6 h HBO exposure period commenced upon reaching 2 ATA. After exposure, decompression to atmospheric pressure was conducted linearly over 20 min, after which the cell cultures were harvested for downstream assays.

### 2.5 Experimental groups and drug treatment

For the first part of the study, experiments were designed to investigate the effects of CYP1A deficiency on HBO-induced acute lung injury (ALI). Mice were randomly assigned to four groups: (a) CYP1A^+/+^ group: CYP1A^+/+^ mice were housed under normobaric air conditions. (b) CYP1A^−/−^ group: CYP1A^−/−^ mice were housed under normobaric air conditions. (c) HBO + CYP1A^+/+^ group: CYP1A^+/+^ mice were exposed to HBO at 2ATA for 6 h. (d) HBO + CYP1A^−/−^ group: CYP1A^−/−^ mice were exposed to HBO at 2ATA for 6 h. Lung tissues were collected for quantitative real-time RT-PCR, immunofluorescence, Western blot analysis, and H&E staining.

For the second part of the study, experiments were conducted to evaluate the effects of the CYP1A inducer β-naphthoflavone (BNF, N865991, Macklin, Shanghai, China) on HBO-induced ALI. BNF was dissolved in corn oil. Mice were randomly assigned to four groups: (a) Control group: Mice housed under normobaric air conditions received daily intraperitoneal injections of corn oil for three consecutive days. (b) BNF group: Mice housed under normobaric air conditions received daily intraperitoneal injections of BNF at a dose of 100 mg/kg/day [24] for three consecutive days. (c) HBO group: Mice received daily intraperitoneal injections of corn oil for three days and were subsequently exposed to HBO at 2ATA for 6 h. (d) HBO + BNF group: Mice received daily intraperitoneal injections of BNF at a dose of 100 mg/kg/day for three days and were subsequently exposed to HBO at 2ATA for 6 h. The BNF dosage was selected based on previous studies [24,25] and our preliminary experiments. Lung tissues were collected for quantitative real-time RT-PCR, immunofluorescence, Western blot analysis, and H&E staining.

For the third part of the study, experiments were conducted to evaluate the effects of the CYP1A inducer BNF on HBO-induced ALI in CYP1A deficient mice. Mice were randomly assigned to four groups: (a) HBO + CYP1A^+/+^ group: CYP1A^+/+^ mice received daily intraperitoneal injections of corn oil for three days and were subsequently exposed to HBO at 2ATA for 6 h. (b) HBO + CYP1A^+/+^ + BNF group: CYP1A^+/+^ mice received daily intraperitoneal injections of BNF at a dose of 100 mg/kg/day for three consecutive days and were subsequently exposed to HBO at 2ATA for 6 h. (c) HBO + CYP1A^−/−^ group: CYP1A^−/−^ mice received daily intraperitoneal injections of corn oil for three days and were subsequently exposed to HBO at 2ATA for 6 h. (d) HBO + CYP1A^−/−^ + BNF group: CYP1A^−/−^ mice received daily intraperitoneal injections of BNF at a dose of 100 mg/kg/day for three consecutive days and were subsequently exposed to HBO at 2ATA for 6 h. Lung tissues were collected for quantitative real-time RT-PCR, immunofluorescence, and H&E staining.

For the fourth part of the study, we aimed to determine whether systemic administration of the HSF1 inhibitor KRIBB11 affected the protective effect of BNF against HBO-induced ALI. KRIBB11 (HY-100872, MCE, Shanghai, China) was dissolved in corn oil. Mice were randomly assigned to the following groups: (a) HBO group: Mice received daily intraperitoneal injections of corn oil for three days and were subsequently exposed to HBO at 2ATA for 6 h. (b) HBO + BNF group: Mice received daily intraperitoneal injections of BNF at a dose of 100 mg/kg/day for three days and were subsequently exposed to HBO at 2ATA for 6 h. (c) HBO + KRIBB11 group: Mice received a single intraperitoneal injection of KRIBB11 at a dose of 50 mg/kg [26] and were exposed to HBO at 2ATA for 6 h one day later. (d) HBO + BNF + KRIBB11 group: Mice received daily intraperitoneal injections of BNF at a dose of 100 mg/kg/day for three days. On the last day of BNF treatment, mice received a single intraperitoneal injection of KRIBB11 at a dose of 50 mg/kg and were exposed to HBO at 2ATA for 6 h one day later. The KRIBB11 dosage was selected based on previous studies [26] and our preliminary experiments. Lung tissues were collected for quantitative real-time RT-PCR, immunofluorescence, Western blot analysis, and H&E staining.

### 2.6 Immunofluorescence analysis

Paraffin-embedded lung tissue sections (4 μm) were deparaffinized and subjected to microwave antigen retrieval in citrate buffer. Following incubation with 3% bovine serum albumin (BSA) for 1 h to block nonspecific binding, the sections were incubated overnight at 4°C with the following primary antibodies: CD31 (1:100, GB11063-2-100, Servicebio), cleaved caspase-3 (1:100, GB11532-100, Servicebio), SDHC (1:100, sc-515102, Santa Cruz), and 8-OHdG (1:100, ab183393, Abcam). After thorough washing, the sections were incubated with Alexa Fluor® 488- and 594-conjugated secondary antibodies (GB25303, Servicebio) in the dark at room temperature for 1 h. Nuclei were counterstained with 4′,6-diamidino-2-phenylindole (DAPI) (G1012, Servicebio). Fluorescence images were captured using a Panoramic MIDI scanner (3D HISTECH, Budapest, Hungary) and analyzed with ImageJ software. The immunofluorescence analysis was performed by researchers who were blinded to the experimental group assignments.

### 2.7 Fluorescein isothiocyanate carboxymethyl (FITC)-dextran flux assay

The transendothelial permeability of FITC-dextran (10 kDa, Sigma-Aldrich) was assessed in vitro using a Transwell assay as previously described [27]. MLVECs were seeded into Transwell inserts (0.4 μm pore size, 12 mm diameter, Corning, NY, USA) and cultured until confluence was achieved. Following treatment, 1 mg/ml FITC-dextran solution was added to the apical chamber and allowed to diffuse for 1 h. Permeability was quantified by measuring FITC-dextran fluorescence in the basolateral chamber using a Synergy™ H1 Multi-Mode Microplate Reader (Agilent, BioTek, USA) at an excitation wavelength of 485 ± 20 nm and an emission wavelength of 528 ± 20 nm.

### 2.8 Cell viability assay

Cell viability was detected using the Cell Counting Kit 8 (CCK-8) kit (Beyotime, China) according to the manufacturer's instructions. The absorbance of each well was measured at 450 nm using the Synergy™ H1 multi-Mode microplate reader (Agilent, BioTek, USA).

### 2.9 Lung tissue histological evaluation

Histopathological changes in lung tissue were examined under an optical microscope following hematoxylin and eosin (H&E) staining. Lung injury scores were independently assessed by two pathologists with expertise in pulmonary pathology. The scoring criteria were adapted from previously established methods [27]: 0 = normal lung architecture; 1 = minimal inflammatory infiltration; 2 = mild to moderate inflammation without structural disruption; 3 = moderate inflammation with thickened alveolar septa; 4 = moderate to severe inflammation with focal nodules or pneumonia-like lesions; 5 = severe inflammation with complete loss of alveolar architecture. Five random microscopic fields were selected from each lung tissue sample, and each field was scored individually. The average score per sample was calculated and used for statistical analysis.

### 2.10 Small interfering RNA (siRNA) transfection

Mouse SDHC-specific siRNA was synthesized by GenePharma (Shanghai, China). The sequences of the mouse SDHC siRNA are as follows: 5′-CUGAUCUACUCGGCUAAGUTT-3′ and 5′-ACUUAGCCGAGUAGAUCAGTT-3’. A scrambled non-targeting siRNA sequence served as the negative control: 5′-UUCUCCGAACGUGUCACGUTT-3′ and 5′-ACGUGACACGUUCGGAGAATT-3’. siRNA transfection into MLVECs was carried out using Lipo8000 (Beyotime, China), according to the manufacturer's instructions.

### 2.11 Western blot

Proteins were extracted from lung tissues or MLVECs using RIPA lysis buffer (Beyotime, China) supplemented with protease and phosphatase inhibitor cocktail (Beyotime, China). Protein concentration was measured using the BCA Protein Assay Kit (Thermo Fisher Scientific, USA). Equal amounts of protein lysates were separated by 10% or 15% SDS-PAGE and subsequently transferred onto polyvinylidene fluoride (PVDF) membranes (Millipore, Massachusetts, USA). The membranes were blocked with 5% non-fat milk to reduce nonspecific binding. Primary antibodies against vascular endothelial (VE)-cadherin (1:1000, ab33168, Abcam), SDHC (1:1000, ab155999, Abcam), HSF1 (1:1000, 21773-1-AP, Abcam), CYP1A1 (1:1000, 13241-1-AP, ProteinTech), CYP1A2 (1:1000, 19936-1-AP, ProteinTech), CYP1B1 (1:1000, 67033-1-Ig, ProteinTech), and β-actin (1:10,000, 66009-1-Ig, ProteinTech) were applied, and the membranes were incubated overnight at 4°C. Following washing, the membranes were incubated with horseradish peroxidase (HRP)-conjugated anti-rabbit (1:1000, SA00001-2, ProteinTech) or anti-mouse (1:1000, SA00001-1, ProteinTech) secondary antibodies for 1 h at room temperature. Immunoreactive protein bands were visualized and analyzed using ImageJ software. Band intensities were normalized to β-actin expression levels for semi-quantitative comparison.

### 2.12 Quantitative real-time RT-PCR

Total RNA was extracted using Trizol reagent (Takara) and then subjected to RT quantitative PCR. The quantitative PCR was performed using SYBR® Premix Ex Taq (Vazyme, China) and Applied Biosystems 7500 Fast Food Safety Real-Time PCR System (Thermo Fisher Scientific, USA). The Ct value of β-actin was used as the control. The primer sequences used in this study are shown in Supplementary Table 1.

### 2.13 RNA sequencing (RNA-seq) and bioinformatics analysis

Total RNA was extracted from lung tissues using TRIzol reagent (Vazyme, China). RNA-seq analysis was performed by OE Biotech (Shanghai, China). The sequencing library was quality-checked using an Agilent 2100 Bioanalyzer and sequenced on the Illumina HiSeq™ 2500 platform. Gene expression levels were quantified as FPKM (fragments per kilobase of exon per million mapped reads). Differentially expressed genes were identified using a Benjamini-Hochberg (BH)-adjusted false discovery rate (FDR) < 0.05 and a fold change ≥1.5 as the significance thresholds. Additionally, gene set enrichment analysis (GSEA, version 4.3.2) was conducted to identify enriched biological pathways.

### 2.14 Dual luciferase assay

The HSF1-responsive reporter plasmid pGL4.41 [luc2P/HSE/Hygro] (Promega, USA) and the internal control plasmid encoding Renilla luciferase were co-transfected into MLVECs with Lipo8000 (Beyotime, China). Twenty-four hours after transfection, cells were exposed to hyperbaric oxygen (2 ATA, 95% ± 5% O_2_) for 6 h in the presence or absence of BNF. After a further 24 h recovery in normoxic culture, cells were lysed and luciferase activities were measured with the Dual-Luciferase® Reporter Assay System (Promega, USA). Firefly luciferase activity was recorded first; Stop & Glo® reagent was then added to quench Firefly and initiate Renilla luminescence. Data are expressed as the Firefly/Renilla luciferase activity ratio (F/R) and normalized to the untreated control group.

### 2.15 Isolation of mitochondria

Mitochondrial isolation was performed using the Mitochondria Fractionation Kit (C3606, Beyotime, China) as previously described [28]. In brief, lung tissues were rapidly excised and rinsed in ice-cold PBS to remove residual blood. The tissues were then minced and washed with the isolation medium, followed by homogenization to prepare 10% (w/v) tissue homogenates. The homogenate was centrifuged at 600 × g at 4°C for 5 min to pellet nuclei and cellular debris. The resulting supernatant was further centrifuged at 10,000 × g for 10 min. Finally, the mitochondrial pellet was resuspended in mitochondrial storage solution.

### 2.16 Detection of mitochondrial superoxide production

MitoSOX (M36008, Thermo Fisher Scientific, USA) is a cell-permeant fluorescent probe that specifically accumulates in mitochondria and emits red fluorescence upon oxidation by superoxide [29]. MitoSOX was freshly dissolved in DMSO immediately before use and subsequently applied to MLVECs or isolated mitochondria at a final concentration of 5 μM. MLVECs stained with MitoSOX™ were counterstained with DAPI for 5 min to label nuclei. Fluorescence images were captured using a Biotek Cytation 5 (Biotek, USA) and analyzed with ImageJ software. For MitoSOX™ staining in isolated mitochondria, red fluorescence intensity was measured at an excitation wavelength of 396 nm and an emission wavelength of 580 nm [30] using a Synergy™ H1 Multi-Mode Microplate Reader (Agilent, BioTek, USA).

### 2.17 Assessment of mitochondrial membrane potential loss

Mitochondrial membrane potential was assessed using the fluorescent probe JC-1 according to the instructions provided in the Enhanced Mitochondrial Membrane Potential Assay Kit (C2003S, Beyotime, China). In cells with depolarized mitochondria, JC-1 predominantly exists in its monomeric form and emits green fluorescence [31]. In contrast, in cells with polarized mitochondria, JC-1 forms aggregates that emit red fluorescence. MLVECs or isolated mitochondria were incubated with 10 μM JC-1 for 20 min to allow probe loading. JC-1-stained MLVECs were counterstained with DAPI for 5 min to label nuclei. Fluorescence images were captured using a Biotek Cytation 5 (Biotek, USA) and analyzed with ImageJ software. For JC-1 staining in isolated mitochondria, red fluorescence was measured at an excitation wavelength of 525 nm and emission wavelength of 590 nm, while green fluorescence was detected at an excitation wavelength of 490 nm and emission wavelength of 530 nm using a Synergy™ H1 Multi-Mode Microplate Reader (Agilent, BioTek, USA). Mitochondrial damage was evaluated by calculating the ratio of red to green fluorescence intensity, which reflects the extent of mitochondrial membrane depolarization.

### 2.18 Measurement of ATP concentration and mitochondrial complex II activity

The ATP concentration in MLVECs or isolated mitochondria was measured using the Enhanced ATP Assay Kit (S0027, Beyotime, China) following the manufacturer's instructions. Mitochondrial complex II activity in lung tissues and MLVECs was determined using the Mitochondrial Complex II/Succinate-Coenzyme Q Reductase Activity Assay Kit (BC3235, Solarbio, Beijing, China). Both ATP levels and mitochondrial complex II activity were normalized to protein content based on the BCA assay and expressed as nmol/mg protein and unit/mg protein, respectively.

### 2.19 Measurement of caspase-3 activity

Caspase-3 activity in MLVECs or lung tissues was determined using the Caspase-3 Activity Assay Kit (C1116, Beyotime, China). This assay is based on the principle that caspase-3 catalyzes the cleavage of the chromogenic substrate Ac-DEVD-pNA (acetyl-aspartic acid-valine-aspartic acid p-nitroaniline), resulting in the release of yellow-colored p-nitroaniline (pNA), which can be quantified by measuring absorbance. Briefly, MLVECs or lung tissues were homogenized in lysis buffer and centrifuged at 20,000 × g for 15 min at 4°C. The supernatants were incubated with Ac-DEVD-pNA for 2 h at 37°C. Absorbance was measured at 405 nm, and caspase-3 activity was normalized to protein content and expressed as units of caspase-3 activity per mg of protein, based on the BCA-determined protein concentration.

### 2.20 Chromatin immunoprecipitation (ChIP) assay

ChIP assays were performed using the Pierce™ Magnetic ChIP Kit (Thermo Scientific, Cat. No. 26157) according to the manufacturer's instructions. Briefly, MLVECs (2 × 10^6^) were cross-linked with 1% formaldehyde for 10 min at room temperature, and the reaction was quenched with glycine. The cells were washed with PBS and subjected to lysis and nuclear extraction using Membrane Extraction Buffer supplemented with protease and phosphatase inhibitors. Chromatin was digested with ChIP-grade Micrococcal Nuclease (MNase) for 15 min at 37°C, and the digestion was stopped with MNase Stop Solution. Nuclei were pelleted, resuspended in IP Dilution Buffer, and sonicated to complete lysis. After centrifugation, the supernatant containing sheared chromatin was collected. An aliquot (10%) was saved as total input control. The remaining chromatin was divided into two portions: one was incubated with anti-HSF1 antibody (ab52757, Abcam), and the other with normal rabbit IgG (Beyotime, China) as a negative control, overnight at 4°C. This was followed by incubation with ChIP-grade Protein A/G Magnetic Beads for 2 h at 4°C. Beads were washed sequentially with IP Wash Buffer 1 and IP Wash Buffer 2. Protein–DNA complexes were eluted with IP Elution Buffer at 65°C for 30 min. Cross-linking was then reversed by adding NaCl and Proteinase K, followed by incubation at 65°C for 1.5 h. DNA was purified and subjected to qPCR using the specific primers listed in Supplementary Table 1.

### 2.21 Statistical analysis

All data in this study were expressed as mean ± standard error of the mean (SEM). Statistical analysis was conducted using SPSS 26.0 software. An independent samples *t*-test was applied to compare the experimental data between two groups. One-way or two-way ANOVA was performed to analyze differences among multiple groups, followed by post-hoc multiple comparisons using the LSD or Bonferroni correction. A *p*-value <0.05 was considered statistically significant.

## 3 Results

### 3.1 CYP1A deficiency aggravates HBO-induced acute lung injury

We first examined the expression levels of CYP1 subfamily members in mouse lung tissues and primary cultured mouse pulmonary microvascular endothelial cells (MLVECs) at 2-, 4-, and 6-h following HBO exposure. The results showed that the mRNA and protein expression of CYP1A1 and CYP1A2 in lung tissues from HBO-exposed mice increased significantly in a time-dependent manner compared to control mice, whereas the expression level of CYP1B1 remained nearly unchanged (Fig. 1A and B and Supplemental Fig. S1A–B). These findings were consistent in MLVECs, where CYP1A1 and CYP1A2 mRNA and protein levels also increased significantly in a time-dependent manner after HBO exposure, while CYP1B1 expression remained stable (Fig. 1C and D and Supplemental Fig. S1C–D). These results suggest that HBO exposure selectively upregulates the expression of CYP1A, but not CYP1B1, indicating their potential involvement in the pathogenesis of HBO-induced lung injury.

**Fig. 1:**
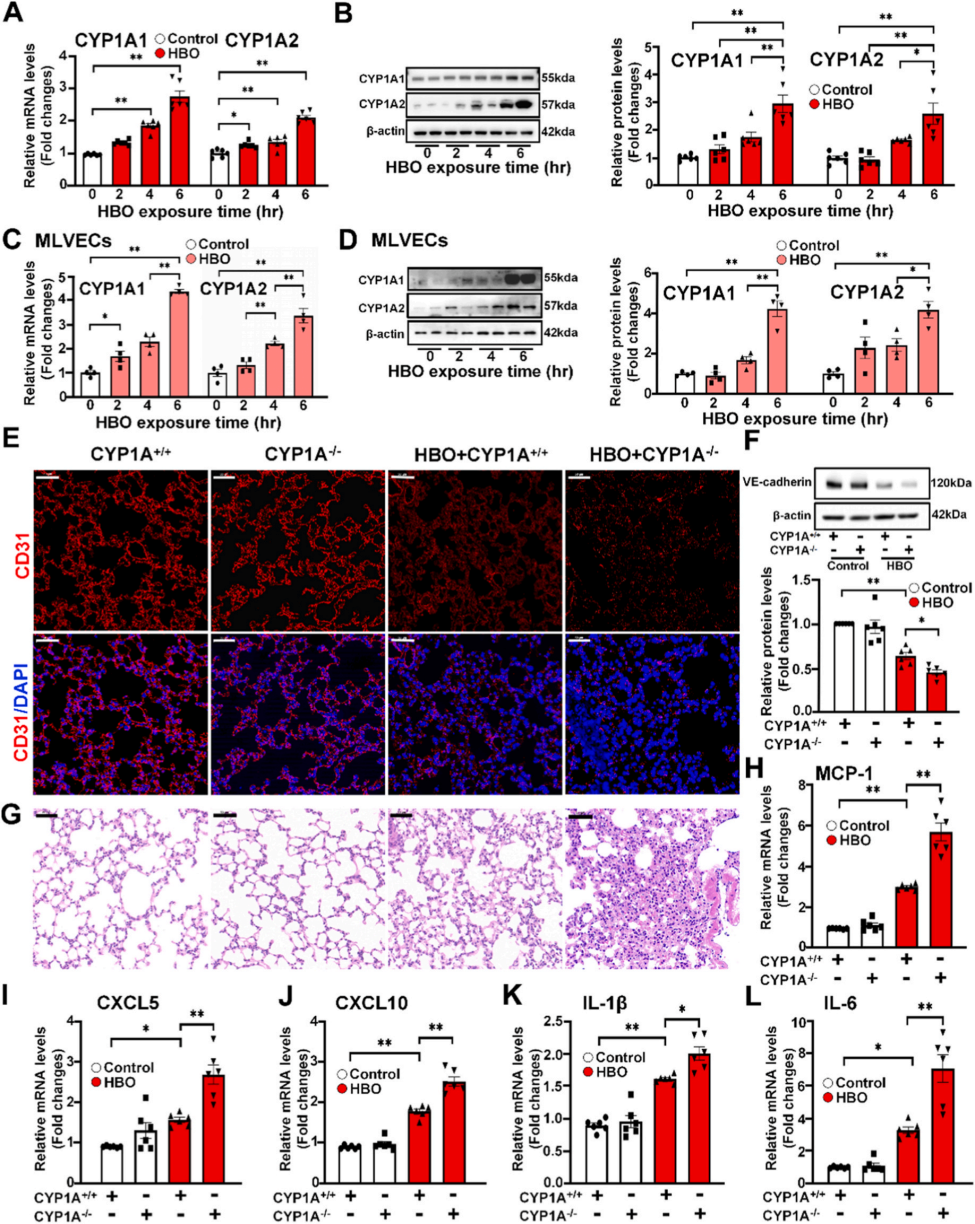
CYP1A gene knockout exacerbates endothelial barrier dysfunction, pathological damage and inflammatory response in the lung tissue of mice exposed to HBO. A, mRNA expression levels of CYP1A1 and CYP1A2 in the lung tissue of mice exposed to HBO for 0, 2, 4, and 6 h were detected by qRT-PCR (n = 6). **B,** Protein expression levels of CYP1A1 and CYP1A2 in the lung tissue of mice exposed to HBO for 0, 2, 4, and 6 h were detected by Western Blot (n = 6), and the corresponding statistical graphs are shown on the right side of the bands. **C,** mRNA expression levels of CYP1A1 and CYP1A2 in MLVECs after exposure to HBO for 0, 2, 4, and 6 h (n = 4). **D,** Protein expression levels of CYP1A1 and CYP1A2 in MLVECs after exposure to HBO for 0, 2, 4, and 6 h (n = 4), and the corresponding statistical graphs are shown on the right side of the bands. **E,** Immunofluorescence shows the expression level of CD31 (red) in the lung tissue of CYP1A gene knockout mice after exposure to HBO. Nuclei were stained with DAPI. Scale bar = 50 μm. **F,** Protein expression levels of VE-cadherin in the lung tissue of CYP1A gene knockout mice exposed to HBO were detected by Western Blot (n = 6), and the related histogram is shown below the bands. **G,** Hematoxylin and eosin staining was used to show the pathological changes in the lung tissue of CYP1A gene knockout mice exposed to HBO. Original magnification, ×400. Scale bar corresponds to 50 μm. **H-L,** mRNA expression levels of chemokines and inflammatory factors MCP-1, CXCL5, CXCL10, IL-1β and IL-6 in the lung tissue of CYP1A gene knockout mice exposed to HBO were detected by qRT-PCR (n = 6). Data are presented as the mean ± SEM. ∗p < 0.05, ∗∗p < 0.01.

To investigate the role of CYP1A in HBO-induced pulmonary endothelial barrier dysfunction and acute lung injury, we generated global double knockout mice for CYP1A1 and CYP1A2 (referred to as CYP1A-deficient mice). As expected, neither mRNA nor protein expression of CYP1A1 and CYP1A2 was detected in the lung tissues of CYP1A-deficient mice (Supplemental Fig. S2A–B). The integrity of the endothelial barrier relies on the endothelial cell-specific adhesion molecules such as VE-cadherin and CD31. Immunofluorescence and Western blot analyses demonstrated that HBO exposure led to significant decreases in the expression levels of CD31 and VE-cadherin, which were further exacerbated by CYP1A deficiency (Fig. 1E–F and Supplemental Fig. S1E). Histological analysis also revealed that HBO-exposed mice exhibited marked pulmonary injury, characterized by increased inflammation, diffuse interstitial edema, alveolar wall thickening, and reduced alveolar airspace, with a lung injury score of 2.83 ± 0.05. Following CYP1A knockout, the severity of pulmonary injury in HBO-exposed mice was further increased, with a lung injury score of 3.37 ± 0.05 (Fig. 1G and Supplemental Fig. S1F). Furthermore, qRT-PCR results showed that the expression levels of chemokines (MCP-1, CXCL5, CXCL10) and pro-inflammatory cytokines (IL-1β, IL-6) were significantly elevated after HBO exposure, and this increase was further enhanced in HBO-exposed CYP1A-deficient mice (Fig. 1H–L). These findings suggest that CYP1A deficiency significantly exacerbates HBO-induced endothelial dysfunction and acute lung injury.

### 3.2 CYP1A inducer ameliorates HBO-induced pulmonary endothelial hyperpermeability and lung injury

We then investigated the effect of the CYP1A inducer β-naphthoflavone (BNF) on endothelial cell injury induced by HBO exposure in MLVECs. As shown in Fig. 2A and Supplemental Fig. S3A, BNF treatment significantly induced the protein expression of both CYP1A1 and CYP1A2 in HBO-exposed MLVECs. HBO exposure caused significant endothelial injury, as evidenced by reduced VE-cadherin expression and decreased cell viability. In addition, we measured the permeability of a confluent MLVEC monolayer to 10-kDa FITC-dextran and found that HBO exposure significantly increased endothelial permeability (Fig. 2B–D). BNF treatment was found to significantly attenuate HBO-induced endothelial damage, as indicated by increased VE-cadherin protein levels and improved cell viability, as well as reduced FITC-dextran leakage (Fig. 2B–D).

**Fig. 2:**
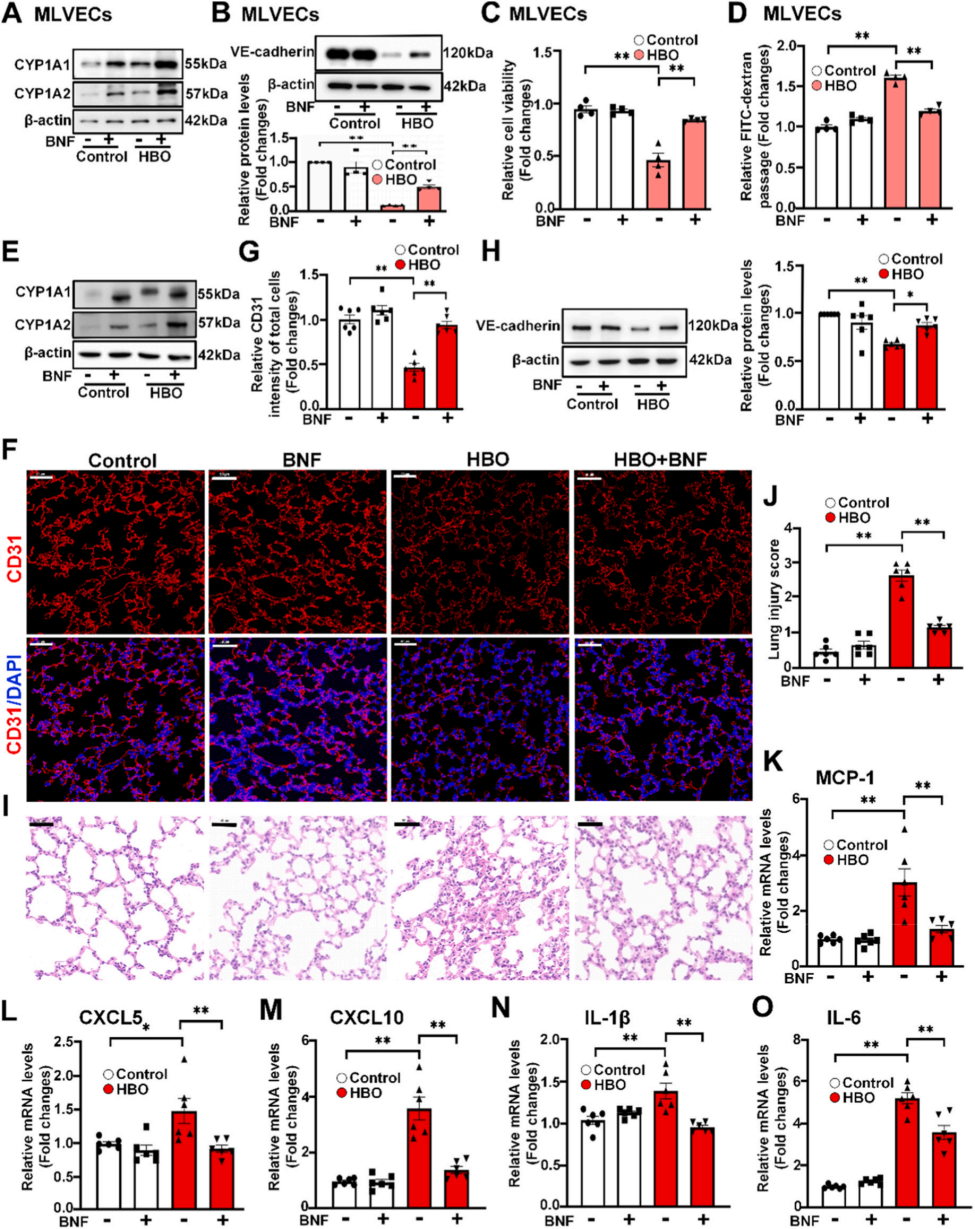
CYP1A inducer improves the endothelial barrier dysfunction of MLVECs and lung tissue after HBO exposure, and alleviates the pathological damage and inflammatory response of lung tissue. A, MLVECs were pretreated with 5 μM CYP1A inducer BNF for 30 min, and the protein expression levels of CYP1A1 and CYP1A2 in HBO-exposed MLVECs after CYP1A inducer treatment were detected by Western Blot (n = 4). **B,** Protein expression level of VE-cadherin in HBO-exposed MLVECs after CYP1A inducer treatment was detected by Western Blot (n = 4), and the corresponding histogram is shown below the band. **C,** Cell viability of MLVECs was detected by CCK-8 (n = 4). **D,** Endothelial cell permeability was determined by FITC-dextran flux assay (n = 4). **E,** Protein expression levels of CYP1A1 and CYP1A2 in the lung tissue of HBO-exposed mice after CYP1A inducer treatment were detected by Western Blot (n = 6). **F,** Immunofluorescence shows the expression level of CD31 (red) in the lung tissue of HBO-exposed mice after CYP1A inducer treatment. The cell nuclei were stained with DAPI. Scale bar = 50 μm. **G,** Fluorescence intensity of CD31 relative to the total cell number is shown in a histogram (n = 6). **H,** Protein expression level of VE-cadherin in the lung tissue of HBO-exposed mice after CYP1A inducer treatment was detected by Western Blot (n = 6), and the corresponding histogram is shown on the right side of the band. **I,** Hematoxylin and eosin staining of lung tissue was used to show the pathological changes in the lung tissue of HBO-exposed mice treated with CYP1A inducers. Original magnification, ×400. Scale bar = 50 μm. **J,** The histogram of lung tissue pathological injury score (n = 6). **K–O,** mRNA expression levels of chemokines and inflammatory factors MCP-1, CXCL5, CXCL10, IL-1β and IL-6 in the lung tissue of HBO-exposed mice after CYP1A inducer treatment were detected by qRT-PCR (n = 6). Data are presented as the mean ± SEM. ∗p < 0.05, ∗∗p < 0.01.

Next, we explored the effect of the CYP1A inducer BNF on HBO-induced pulmonary endothelial dysfunction and lung injury in vivo. As expected, BNF significantly elevated pulmonary expression levels of CYP1A1 and CYP1A2 in HBO-exposed mice (Fig. 2E and Supplemental Fig. S3B). Immunofluorescence and Western blot analyses demonstrated that treatment with the CYP1A inducer significantly reversed HBO-induced decreases in CD31 and VE-cadherin expression (Fig. 2F–H). Histological analysis revealed that the lung injury score in CYP1A inducer-treated mice

was significantly lower than that in vehicle-treated mice following HBO exposure (Fig. 2I and J). Furthermore, CYP1A inducer treatment significantly suppressed HBO-induced expression of chemokines and pro-inflammatory cytokines in lung tissues (Fig. 2K–O).

We then assessed whether the protective effects of the CYP1A inducer BNF against HBO-induced lung injury are strictly dependent on CYP1A by evaluating BNF treatment in CYP1A-deficient mice. In contrast to the robust protection observed in wild-type littermates, BNF conferred modest yet statistically significant protection in CYP1A-deficient mice. This was evidenced by partial restoration of pulmonary CD31 protein expression, a measurable reduction in histopathological lung injury scores, and attenuated mRNA expression of key inflammatory mediators, including the chemokines MCP-1, CXCL5, and CXCL10, and the cytokines IL-1β and IL-6 (Supplemental Fig. S4). These findings indicate that while CYP1A is the principal mediator of BNF's protective action, residual protection persists in its absence.

Taken together, the in vitro and in vivo results demonstrate that the CYP1A inducer can improve pulmonary endothelial barrier dysfunction and acute lung injury in mice.

### 3.3 HSF1 activation ameliorates mitochondrial oxidative stress, mitochondrial dysfunction and cell apoptosis through a SDHC-dependent pathway, thus mediating the protective effects of CYP1A against HBO-induced pulmonary endothelial hyperpermeability

To explore the mechanism by which CYP1A protects against HBO-induced endothelial injury, we conducted RNA-Seq analysis on the lung tissues of CYP1A deficient mice or CYP1A inducer BNF-treated mice exposed to HBO. We identified 61 upregulated DEGs and 85 downregulated DEGs in the lung tissues of HBO-exposed CYP1A deficient mice versus HBO-exposed control mice (Fig. 3A). Following Hallmark, Gene Ontology (GO), and Reactome pathway analysis of GSEA, gene sets associated with endothelial barrier, cell adhesion, angiogenesis, and cell proliferation were negatively enriched in HBO-exposed CYP1A deficient mice (Supplemental Fig. S5). In the lung tissues of BNF-treated, HBO-exposed mice versus vehicle-treated, HBO-exposed mice, 304 upregulated DEGs and 546 downregulated DEGs were identified (Fig. 3B). GSEA analysis showed that gene sets associated with mitochondrial function, aerobic respiration, detoxification of ROS, DNA repair, and cell cycle were significantly enriched in BNF-treated, HBO-exposed mice, whereas gene sets associated with inflammatory responses and cell chemotaxis were significantly enriched in vehicle-treated, HBO-exposed mice (Supplemental Fig. S6).

**Fig. 3:**
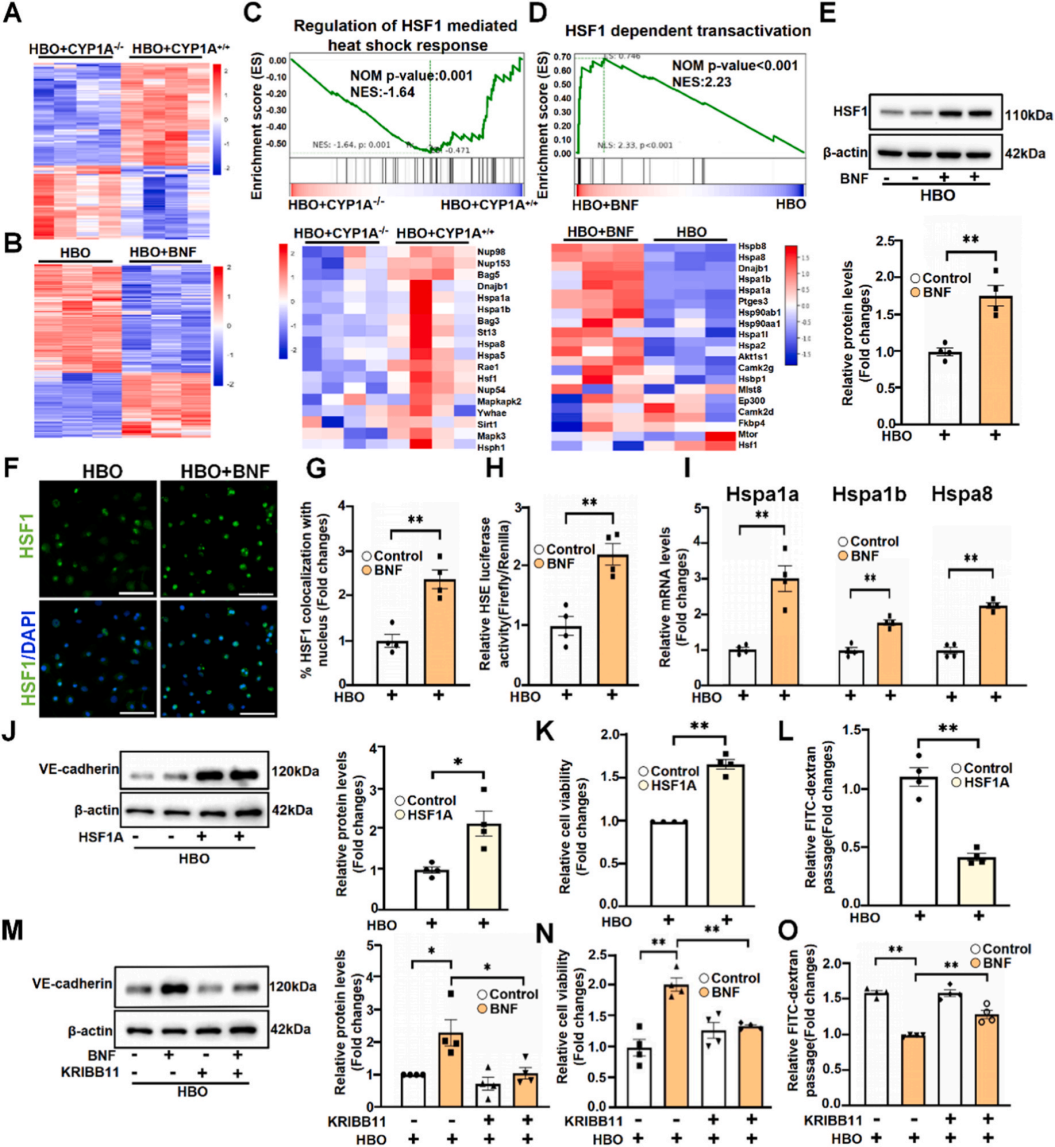
HSF1 activation mediates the protective effects of CYP1A against HBO-induced pulmonary endothelial hyperpermeabi**lity. A-B,** Heatmap of differentially expressed genes in the lung tissues of mice with acute lung injury induced by hyperbaric oxygen and treated with CYP1A gene knockout and CYP1A inducer compared to the lung tissues of mice exposed to HBO. Red indicates upregulated genes, and blue indicates downregulated genes. **C-D,** GSEA revealed that the HSF1 signaling pathway was negatively enriched in CYP1A gene knockout **(C)** and positively enriched after CYP1A inducer treatment **(D)**. Below are the heat maps of the differentially expressed genes of the HSF1 signaling pathway in the lung tissues of CYP1A gene knockout mice **(C)** and CYP1A inducer mice **(D)**. Red indicates upregulated genes, and blue indicates downregulated genes. **E,** After 30 min of 5 μM CYP1A inducer BNF pretreatment, the protein expression level of HSF1 in HBO-exposed MLVECs after CYP1A inducer treatment was detected by Western Blot (n = 4), and the corresponding statistical graph was shown below the band. **F,** After 30 min of 5 μM CYP1A inducer BNF pretreatment, the nuclear translocation of HSF1 in HBO-exposed MLVECs was immunofluorescence labeled (green). The cell nucleus was stained with DAPI. Scale bar = 50 μm. **G,** The number of cells with overlapping HSF1 (green) and DAPI was presented in a histogram (n = 4). **H,** The dual luciferase reporter gene experiment detected the activation effect of BNF on the HSE binding element (n = 4). **I,** The mRNA expression levels of Hspa1a, Hspa1b and Hspa8 of the HSF1 signaling pathway target genes in HBO-exposed MLVECs after CYP1A inducer treatment were detected by qRT-PCR (n = 4). **J,** After 50 μM HSF1 agonist HSF1A treatment of MLVECs, the protein expression level of VE-cadherin in HBO-exposed MLVECs after HSF1A treatment was detected by Western Blot (n = 4), and the corresponding statistical graph was shown on the right side of the band. **K,** The cell viability of MLVECs was detected by CCK-8 (n = 4). **L,** The permeability of endothelial cells was determined by FITC-dextran flux assay (n = 4). **M,** After 30 min of 5 μM BNF and 2 μM HSF1 inhibitor KRIBB11 pretreatment of MLVECs, the protein expression level of VE-cadherin in HBO-exposed MLVECs was detected by Western Blot (n = 4), and the corresponding statistical graph was shown on the right side. **N,** The cell viability was detected by CCK-8 (n = 4). **O,** The permeability of endothelial cells was determined by FITC-dextran flux assay (n = 4). Data are presented as the mean ± SEM. ∗p < 0.05, ∗∗p < 0.01.

Intriguingly, it was found that the HSF1 signaling mediated pathways, which were negatively enriched in the lung tissues of HBO-exposed CYP1A deficient mice (Fig. 3C), were positively enriched in the lung tissues of BNF-treated, HBO-exposed mice (Fig. 3D). From RNA-seq data, target genes of the transcriptional factor HSF1 targets were profoundly downregulated in lung tissues from HBO-exposed CYP1A deficient mice, whereas were upregulated in BNF-treated, HBO-exposed mice (e.g., *Dnajb1, Hspa1l, Hspa1a, Hspa1b, Hspa8*) (Fig. 3C–D). Western blot and immunofluorescence staining results demonstrated that treatment with CYP1A inducer BNF resulted in significant increases in HSF1 expression (Fig. 3E) and nuclear translocation (Fig. 3F–G) in MLVECs exposed to HBO, respectively. Using a HSF1 luciferase reporter gene, we found that CYP1A inducer BNF significantly increased luciferase activity of the HSF1 reporter gene, indicating the transcriptional activation of HSF1 (Fig. 3H). Furthermore, the mRNA levels of HSF1 target genes Hspa1a, Hspa1b, and Hspa8 were significantly induced by BNF treatment in MLVECs (Fig. 3I). These findings indicate that treatment with CYP1A inducer leads to the activation of HSF1 signaling pathway in pulmonary endothelial cells.

As shown in Fig. 3, we found that treatment with HSF1 agonist HSF1A mimicked the protective effects of CYP1A inducer against HBO-induced endothelial damage, as indicated by increased VE-cadherin protein levels and improved cell viability, as well as reduced FITC-dextran leakage (Fig. 3J–L). In contrast, BNF-induced increases in VE-cadherin expression and cell viability were entirely abolished by HSF1 inhibitor KRIBB11 in HBO-exposed MLVECs (Fig. 3M–N). HSF1 inhibitor also reversed the inhibitory effect of BNF on HBO-induced FITC-dextran leakage (Fig. 3O). Collectively, these results indicate that activation of HSF1 signaling pathway contributes to the protective effect of the CYP1A inducer against the pulmonary endothelial barrier dysfunction induced by hyperbaric oxygen.

To further clarify how the HSF1 signaling pathway mediates the protective effect of CYP1A on endothelial cells, as indicated by the GSEA analysis, an additional GSEA analysis was performed. The results demonstrated that gene sets associated with mitochondrial function and the detoxification of ROS were positively enriched in the lung tissues of BNF-treated, HBO-exposed mice, as opposed to those vehicle-treated, HBO-exposed mice (Supplemental Fig. S6). Previous studies have shown that activation of HSF1 can inhibit mitochondrial oxidative stress and thereby alleviate mitochondrial pathway-triggered cell apoptosis [[32], [33], [34]]. We then examined the effect of CYP1A inducer and HSF1 modulators on HBO-induced changes in mitochondrial oxidative stress and mitochondrial functions in MLVECs. As shown in Fig. 4, MitoSOX and JC-1 staining showed significant increases in mitochondrial superoxide production and JC-1 monomers in HBO-exposed endothelial cells, which was significantly suppressed by HSF1A treatment, indicating that activation of HSF1 alleviates mitochondrial oxidative stress and restored mitochondrial membrane potential in HBO-exposed pulmonary endothelial cells exposed to HBO (Fig. 4A–D and Supplemental Fig. S7A–B). Additionally, HBO-induced reduction in ATP production was also reversed by treatment with HSF1 agonist (Fig. 4E). In contrast, BNF-induced decreases in mitochondrial superoxide production and JC-1 monomers, as well as BNF-induced increase in ATP production were profoundly reversed by HSF1 inhibitor KRIBB11 in HBO-exposed MLVECs (Fig. 4F–J and Supplemental Fig. S8A–B).

**Fig. 4:**
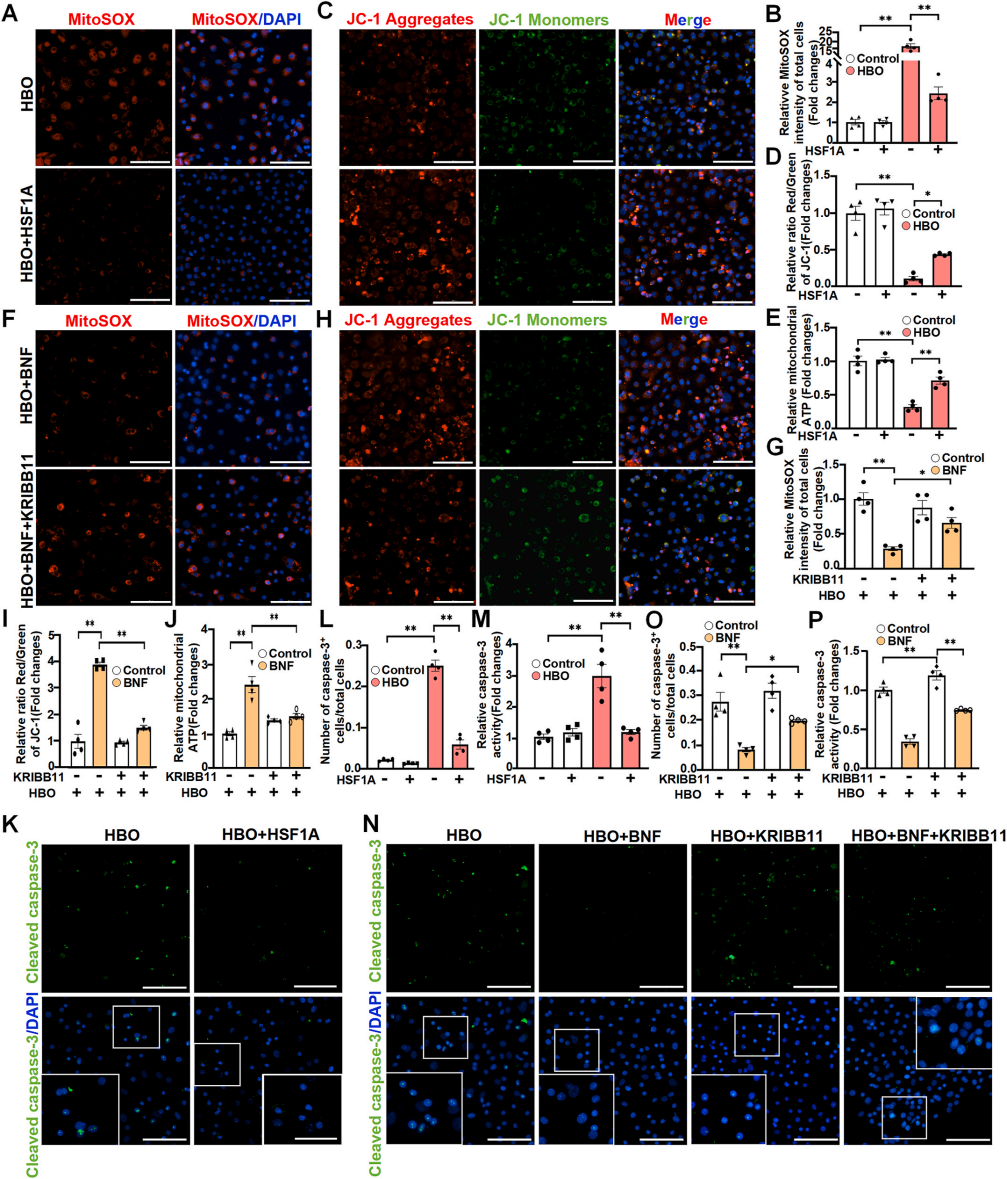
HSF1 activation mediates the protective effects of CYP1A against HBO-induced mitochondrial oxidative stress, mitochondrial dysfunction and cell apoptosis in pulmonary endothe**lial cells**. **A,** Pre-treat MLVECs with 50 μM HSF1 agonist HSF1A for 30 min, and then use MitoSOX probe to stain superoxide (red) after HBO exposure. The cell nucleus is stained with DAPI. Scale bar = 50 μm. **B,** The fluorescence intensity of MitoSOX-labeled superoxide is represented by a histogram (n = 4). **C,** MLVECs were pretreated with 50 μM HSF1 agonist HSF1A for 30 min, followed by exposure to HBO. JC-1 staining was performed, with red indicating JC-1 aggregates and green indicating JC-1 monomers. The cell nuclei were stained with DAPI. Scale bar = 50 μm. **D,** Membrane potential was reflected by the ratio of fluorescence intensity of JC-1 aggregates (red) to that of JC-1 monomers (green) (n = 4). **E,** ATP content was detected after HBO exposure in MLVECs pretreated with 50 μM HSF1 agonist HSF1A for 30 min (n = 4). **F-G,** MLVECs were pretreated with 5 μM BNF and 2 μM KRIBB11 for 30 min, followed by HBO exposure. MitoSOX probe staining was used to detect superoxide (red) after HBO exposure. The fluorescence intensity of MitoSOX-labeled superoxide was represented by a histogram (H) (n = 4). **H–I,** Pre-treat MLVECs with 5 μM BNF and 2 μM KRIBB11 for 30 min, and then use MitoSOX probe to stain superoxide (red) after HBO exposure. The cell nucleus is stained with DAPI. Scale bar = 50 μm **(H)**. The fluorescence intensity ratio of JC-1 aggregates (red) to monomers (green) is represented by a histogram **(I)** (n = 4). **J,** Pre-treat MLVECs with 5 μM BNF and 2 μM KRIBB11 for 30 min, and then detect ATP content after HBO exposure (n = 4). **K,** After treating MLVECs with 50 μM HSF1 agonist HSF1A, immunofluorescence labels the expression level of Cleaved caspase-3 (green) in HBO-exposed MLVECs. The cell nucleus is stained with DAPI. Scale bar = 50 μm. **L,** The number of cells with overlapping Cleaved caspase-3 (green) and DAPI is shown by a histogram (n = 4). **M,** Detection of caspase-3 activity in HBO-exposed MLVECs after HSF1 agonist treatment (n = 4). **N,** Pre-treat MLVECs with 5 μM BNF and 2 μM HSF1 inhibitor KRIBB11 for 30 min, and then immunofluorescence labels the expression level of Cleaved caspase-3 (green) in HBO-exposed MLVECs. The cell nucleus is stained with DAPI. Scale bar = 50 μm. **O,** The number of cells with overlapping Cleaved caspase-3 (green) and DAPI is shown by a histogram (n = 4). **P,** Detection of caspase-3 activity in HBO-exposed MLVECs (n = 4). Data are presented as the mean ± SEM. ∗p < 0.05, ∗∗p < 0.01.

Next, we observed the effect of CYP1A inducer and HSF1 modulators on HBO-induced cell apoptosis in MLVECs. As shown in Fig. 4, either HSF1A or CYP1A inducer BNF treatment significantly decreased cleaved caspase-3-positive endothelial cell numbers and caspase-3 activity in HBO-exposed MLVECs, indicating the anti-apoptotic effects of HSF1A and CYP1A inducer (Fig. 4K–P and Supplemental Fig. S7C). However, the anti-apoptotic effects of CYP1A inducer were markedly reversed by treatment with HSF1 inhibitor KRIBB11 (Fig. 4N–P). Taken together, these results indicate that HSF1 activation mediates the protective effect of CYP1A inducer against HBO-induced mitochondrial oxidative stress, mitochondrial dysfunction and cell apoptosis.

We then explored how HSF1 activation regulates mitochondrial functions. GSEA analysis demonstrated that gene sets associated with oxidative phosphorylation were positively enriched in lung tissues of BNF-treated, HBO-exposed mice versus vehicle-treated, HBO-exposed mice (Fig. 5A). Of particular interest, succinate dehydrogenase C (SDHC), a key enzyme of mitochondrial oxidative phosphorylation, was recently identified as a direct target gene of HSF1 [35], and was also observed to be significantly upregulated at the expression level (LogFC −2.38, p < 0.001) in lung tissues of BNF-treated, HBO-exposed mice as compared to vehicle-treated, HBO-exposed mice (Fig. 5A). We found that that treatment with CYP1A inducer BNF resulted in significant increases in mRNA and protein expression of SDHC in MLVECs exposed to HBO (Fig. 5B–C). The potential HSF1-binding sites within the SDHC promoter were then predicted using two complementary, widely validated tools: JASPAR (https://jaspar.genereg.net/) and TFBIND (http://tfbind.hgc.jp/). Two conservative consensus HSF1-binding motifs (−1416 to −1408 bp, and −171 to −161 bp relative to the transcription start site of mouse SDHC) were found in the proximal region of both human and mouse SDHC promoter (Fig. 5D). Subsequent chromatin immunoprecipitation followed by quantitative PCR (ChIP-qPCR) in MLVECs exposed to HBO revealed that BNF treatment significantly increased the binding of HSF1 to two consensus HSF1-binding motifs on the SDHC promoter, which was largely suppressed by HSF1 inhibitor KRIBB11 (Fig. 5D). These results demonstrate that HSF1 directly binds to the SDHC promoter to transcriptionally activate its expression. Additionally, immunofluorescence staining results demonstrated that BNF-induced increases in SDHC were significantly inhibited by HSF1 inhibitor KRIBB11 (Fig. 5E and Supplemental Fig. S9). SDHC is one of the subunits of complex II in the mitochondrial electron transport chain. It was found that BNF treatment led to enhanced activity of mitochondrial complex II in MLVECs exposed to HBO, which was profoundly blocked by either KRIBB11 treatment or siRNA-mediated knockdown of SDHC (Fig. 5F–H and Supplemental Fig. S10). Collectively, these results suggest that CYP1A inducer stimulates SDHC expression, thus increasing the activity of mitochondrial complex II in a HSF1-dependent manner.

**Fig. 5:**
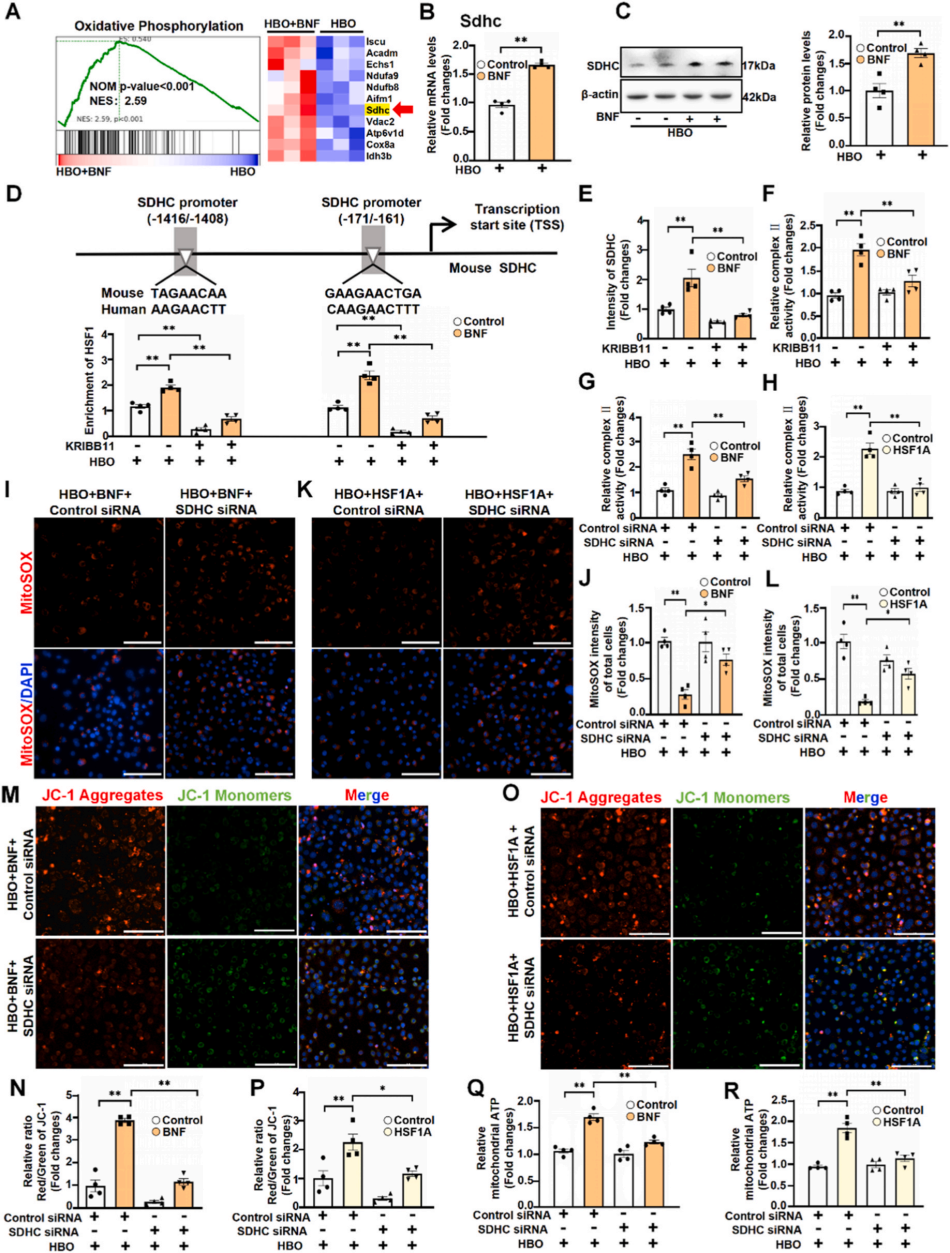
HSF1 activation mediates the protective effects of CYP1A against HBO-induced mitochondrial oxidative stress and mitochondrial dysfunction and through a SDHC-dependent **manner. A,** In the GSEA results, the oxidative phosphorylation pathway shows positive enrichment in the lung tissues of HBO-exposed mice treated with CYP1A inducer. The right side is a heatmap of differentially expressed genes, with red indicating upregulated genes and blue indicating downregulated genes. **B,** 5 μM CYP1A inducer BNF pretreatment of MLVECs for 30 min, and qRT-PCR detection of Sdhc mRNA expression levels in MLVECs after HBO exposure (n = 4). **C,** after 5 μM BNF pretreatment, Western Blot detection of SDHC protein expression levels in HBO-exposed MLVECs, and the corresponding statistical graph is shown on the right side (n = 4). **D-F,** MLVECs were pretreated with 5 μM BNF and 2 μM HSF1 inhibitor KRIBB11 for 30 min prior to HBO exposure. **D**, a schematic diagram shows two predicted conservative consensus HSF1-binding motifs in the proximal region of both mouse and human SDHC promoters. Chromatin immunoprecipitation (ChIP) assay showed enrichment of HSF1 on two consensus HSF1-binding motifs in mouse promoter. **E,** immunofluorescence labeling of SDHC. Fluorescence intensity of SDHC is shown in a histogram (n = 4). **F,** detection of mitochondrial complex II activity after HBO exposure (n = 4). **G-H,** After siRNA knockdown of SDHC in MLVECs, pretreatment with 5 μM BNF **(G)** and 50 μM HSF1 agonist HSF1A **(H)** for 30 min, detection of mitochondrial complex II activity after HBO exposure (n = 4). **I-L,** Pretreatment with 5 μM BNF **(I**–**J)** and 50 μM HSF1A **(K**–**L)** of MLVECs, HBO exposure followed by staining of superoxide with MitoSOX probe (red). The cell nucleus is stained with DAPI. Scale bar = 50 μm. MitoSOX-labeled superoxide fluorescence intensity is shown in a histogram (n = 4). **K,** After 50 μM HSF1A pretreatment of MLVECs, HBO exposure followed by staining of superoxide with MitoSOX probe (red). The cell nucleus is stained with DAPI. Scale bar = 50 μm. **L,** MitoSOX-labeled superoxide fluorescence intensity is shown in a histogram (n = 4). **M-P,** Pretreatment with 5 μM BNF **(M**–**N)** and 50 μM HSF1A **(O–P)** of MLVECs, HBO exposure followed by JC-1 staining, red indicates JC-1 aggregates, green indicates JC-1 monomers. The cell nucleus is stained with DAPI. Scale bar = 50 μm. The fluorescence intensity ratio of JC-1 aggregates (red) to monomers (green) reflects membrane potential (n = 4). **Q-R,** After 5 μM BNF **(Q)** and 50 μM HSF1A **(R)** pretreatment of MLVECs for 30 min, detection of ATP content after HBO exposure (n = 4). Data are presented as the mean ± SEM. ∗p < 0.05, ∗∗p < 0.01.

We then examined whether SDHC was involved in the regulation of CYP1A inducer and HSF1 agonist on HBO-induced mitochondrial dysfunction and cell apoptosis. As shown in Fig. 5, we found that either BNF or HSF1A treatment led to significant decreases in mitochondrial superoxide production and JC-1 monomers, as well as increase in ATP production in HBO-exposed MLVECs, which were significantly reversed by SDHC knockdown (Fig. 5I–R and Supplemental Fig. S11). Additionally, SDHC knockdown abolished the anti-apoptotic effects of BNF or HSF1A in HBO-exposed endothelial cells, as evidenced by increased numbers of cleaved caspase-3-positive endothelial cells and caspase-3 activity (Fig. 6A–F).

**Fig. 6:**
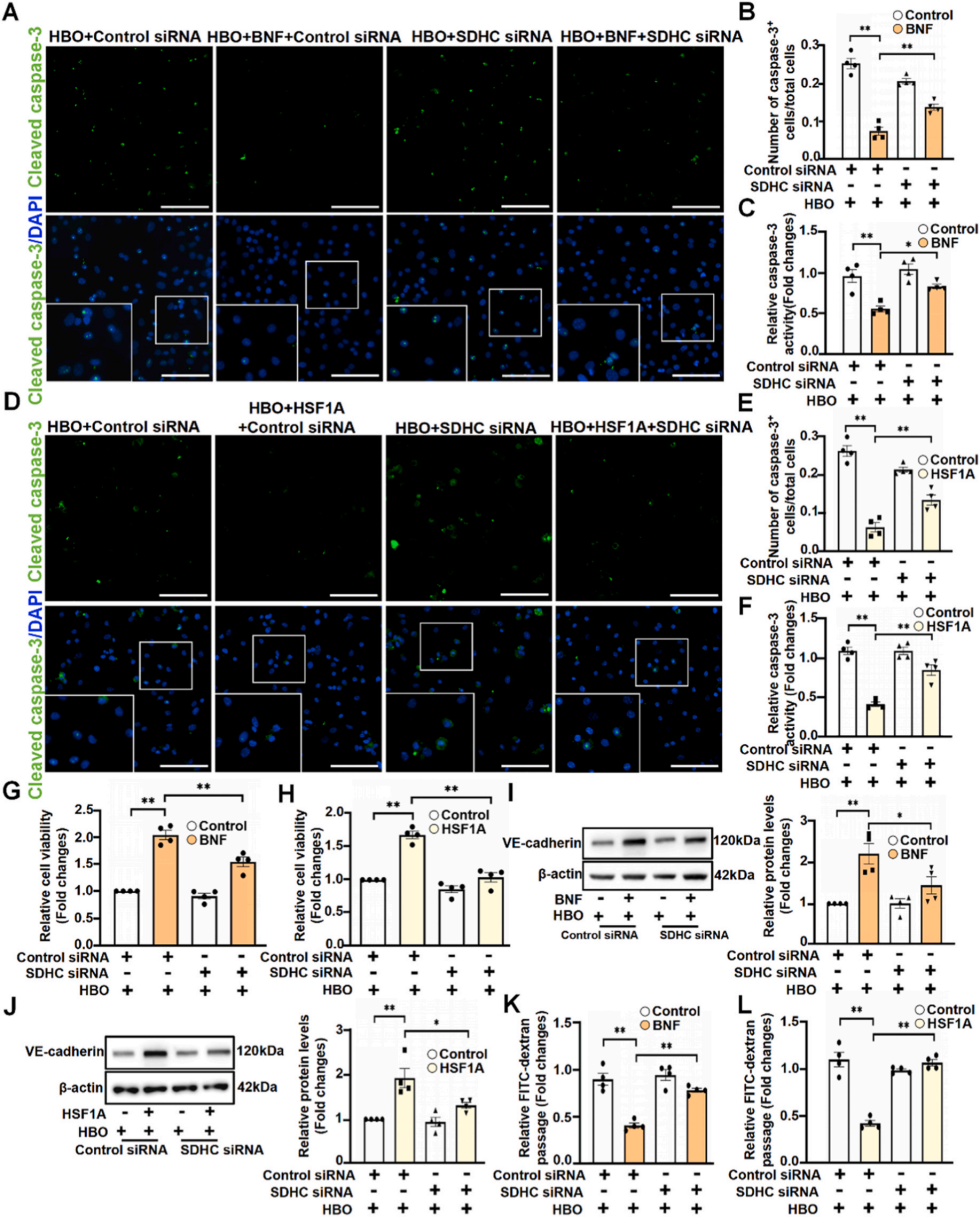
HSF1 activation mediates the protective effects of CYP1A against HBO-induced pulmonary endothelial cell apoptosis and hyperpermeability through a SDHC-dependent **manner. A,** After SDHC knockdown, MLVECs were pretreated with 5 μM BNF and immunofluorescence was used to label the expression level of Cleaved caspase-3 (green) in HBO-exposed MLVECs. The cell nuclei were stained with DAPI. Scale bar = 50 μm. **B,** The number of cells with Cleaved caspase-3 (green) overlapping with DAPI is shown in a histogram (n = 4). **C,** After SDHC knockdown, MLVECs were pretreated with 5 μM BNF and caspase-3 activity was detected after HBO exposure (n = 4). **D,** After SDHC knockdown, MLVECs were pretreated with 50 μM HSF1A and Cleaved caspase-3 (green) expression level was labeled in HBO-exposed MLVECs by immunofluorescence. The cell nuclei were stained with DAPI. Scale bar = 50 μm. **E,** The number of cells with Cleaved caspase-3 (green) overlapping with DAPI is shown in a histogram (n = 4). **F,** After SDHC knockdown, MLVECs were pretreated with 50 μM HSF1A and caspase-3 activity was detected after HBO exposure (n = 4). **G-H,** After knocking down SDHC in MLVECs using siRNA, MLVECs were pretreated with 5 μM BNF **(G)** and 50 μM HSF1A **(H)** for 30 min, and cell viability was detected after HBO exposure (n = 4). **I-J,** After SDHC knockdown, MLVECs were pretreated with 5 μM BNF **(I)** and 50 μM HSF1A **(J)**, and VE-cadherin expression level was detected after HBO exposure. The corresponding histograms are shown on the right side of the band (n = 4). **K-L,** After SDHC knockdown, MLVECs were pretreated with 5 μM BNF **(K)** and 50 μM HSF1A **(L)**, and endothelial cell permeability was determined by FITC-polyethylene glycol flux assay after HBO exposure (n = 4). Data are presented as the mean ± SEM. ∗p < 0.05, ∗∗p < 0.01.

As shown in Fig. 6G–L, we found that HSF1A treatment also mirrored the protective effects of CYP1A inducer BNF on HBO-induced endothelial injury, as evidenced by increased cell viability and VE-cadherin expression, as well as decreased FITC-dextran leakage in MLVECs exposed to HBO exposure. SDHC knockdown significantly decreased cell viability and VE-cadherin expression, meanwhile increased FITC-dextran leakage in HBO-exposed endothelial cells treated by either BNF or HSF1A, indicating that the endothelial cell-protective effects of BNF or HSF1A in HBO-exposed endothelial cells were also significantly attenuated by SDHC knockdown.

Taken together, our results indicate that HSF1 activation ameliorates mitochondrial oxidative stress, mitochondrial dysfunction and cell apoptosis through a SDHC-dependent pathway, thus mediating the protective effects of CYP1A against HBO-induced pulmonary endothelial hyperpermeability.

### 3.4 HSF1 inhibitor decreases SDHC expression and abolishes the protective effects of CYP1A inducer against HBO-induced mitochondrial oxidative stress, mitochondrial dysfunction and endothelial cell apoptosis in lung tissues

In the HBO-exposed mouse model, we found that treatment with CYP1A inducer BNF also significantly increased HSF1 protein expression and the mRNA levels of the HSF1 target genes Hspa1a, Hspa1b, and Hspa8 in lung tissues (Supplemental Fig. S12). As shown in Fig. 7, we found that BNF treatment resulted in profound increases in SDHC expression and mitochondrial complex II activity in lung tissues of HBO-exposed mice, both of which were significantly suppressed by HSF1 inhibitor KRIBB11 (Fig. 7A–C).

**Fig. 7:**
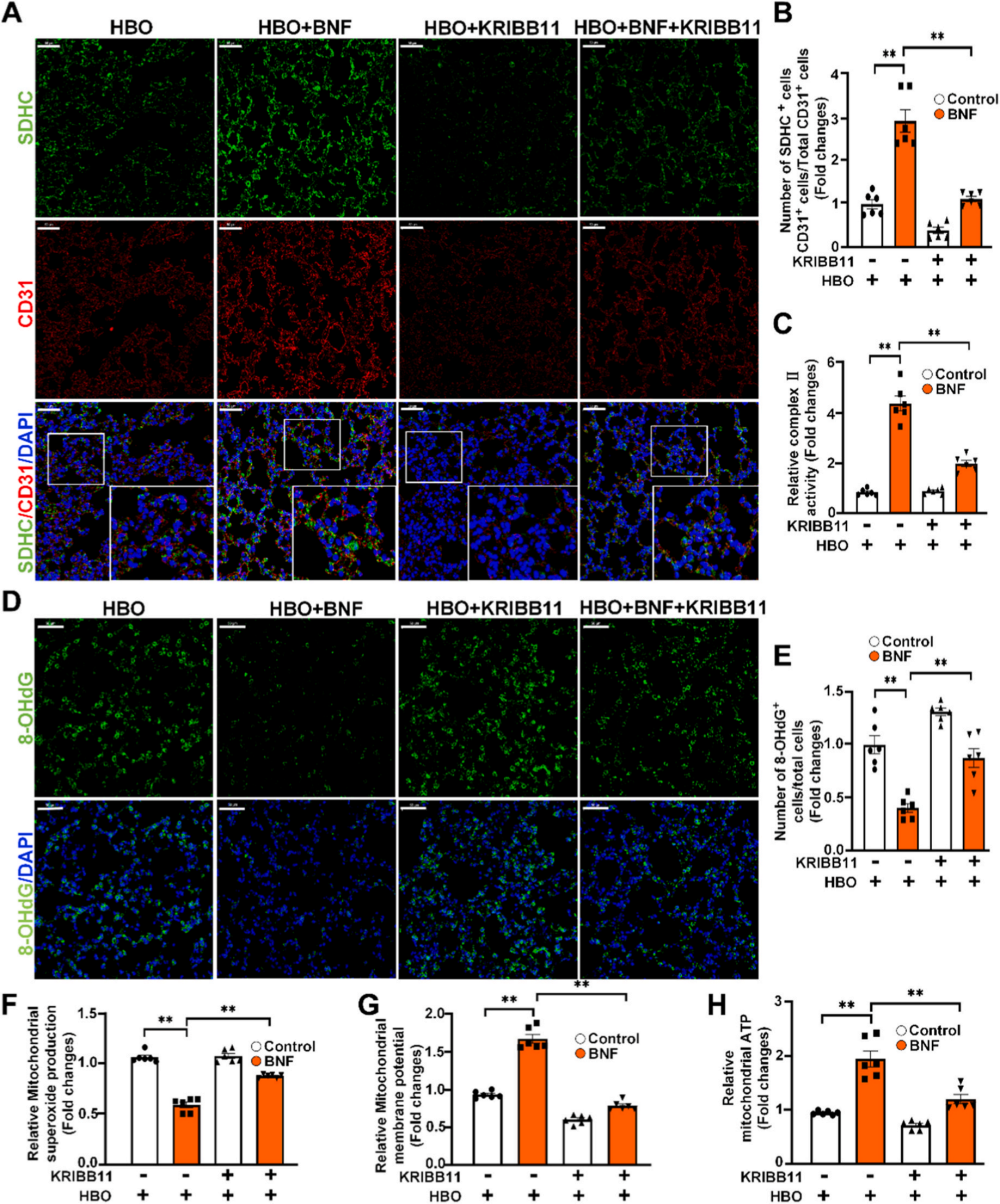
HSF1 inhibitor decreases SDHC expression and abolishes the protective effects of CYP1A inducer against HBO-induced mitochondrial oxidative stress and mitochondrial dysfunction in lung t**issues. A,** After BNF treatment of lung tissue from HBO-exposed mice and KRIBB11 treatment of the lung tissue of HBO-exposed mice, the expression levels of SDHC (green) and endothelial cell marker CD31 (red) were immunofluorescently labeled. The cell nuclei were stained with DAPI. Scale bar = 50 μm. **B,** The proportion of cells co-positive for SDHC and CD31 among total CD31-positive cells was represented by a histogram (n = 6). **C,** The activity of mitochondrial complex II after BNF and KRIBB11 treatment of lung tissue from HBO-exposed mice (n = 6). **D,** The expression level of 8-OHdG (green) in the lung tissue of mice pretreated with BNF and KRIBB11 and exposed to HBO was immunofluorescently labeled. The cell nuclei were stained with DAPI (blue). Scale bar = 50 μm. **E,** The number of cells with overlapping 8-OHdG (green) and DAPI (blue) was presented by a histogram (n = 6). **F,** The fluorescence intensity of MitoSOX in lung mitochondria after BNF and KRIBB11 treatment of HBO-exposed mice (n = 6). **G,** The ratio of fluorescence intensity of JC-1 aggregates (red) and monomers (green) in lung mitochondria after BNF and KRIBB11 treatment of HBO-exposed mice (n = 6). **H,** The ATP content in lung mitochondria after BNF and KRIBB11 treatment of HBO-exposed mice (n = 6). Data are presented as the mean ± SEM. ∗p < 0.05, ∗∗p < 0.01.

We then investigated the effect of HSF1 inhibitor on the protective effects of CYP1A inducer against HBO-induced mitochondrial oxidative stress and dysfunction in lung tissues. 8-OHdG immunoreactivity was first measured as the index of oxidative DNA damage. As shown in Fig. 7D–E, treatment with CYP1A inducer BNF significantly decreased 8-OHdG-positive cells in lung tissues of HBO-exposed mice, which were largely increased by HSF1 inhibitor KRIBB11. Mitochondria were then isolated from lung tissues for assessment of biomarkers of mitochondrial dysfunction.

As shown in Fig. 7F–H, mitochondria isolated from BNF-treated, HBO-exposed mice were found to have a significant decrease in mitochondrial superoxide production, as well as increases in mitochondrial membrane potential and ATP production as compared with vehicle-treated, HBO-exposed mice. Furthermore, the protective effects of CYP1A inducer against HBO-induced mitochondrial oxidative stress and dysfunction in lung tissues were markedly reversed by treatment with HSF1 inhibitor.

We then investigated the effect of HSF1 inhibitor on the protective effects of CYP1A inducer against HBO-induced pulmonary endothelial cell apoptosis and lung injury. As shown in Fig. 8, CYP1A inducer BNF treatment caused significant decreases in caspase-3 activity as well as the percentage of cleaved caspase-3/CD31^+^ cells in total CD31^+^ cells in lung tissues of HBO-exposed mice, which was substantially increased by HSF1 inhibitor KRIBB11 (Fig. 8A–C). In addition, quantitative analysis of the immunofluorescence staining results also demonstrated that HSF1 inhibitor significantly decreased CD31^+^ positive endothelial cells in lung tissues of BNF-treated, HBO-exposed mice (Fig. 8D). In consistent with the results of Fig. 2, BNF treatment led to a significant increase in VE-cadherin expression, as well as decreases in lung injury score and mRNA expressions of chemokines and pro-inflammatory cytokines in lung tissues of HBO-exposed mice, all these protective effects of BNF were abolished by HSF1 inhibitor KRIBB11 (Fig. 8E–L).

**Fig. 8:**
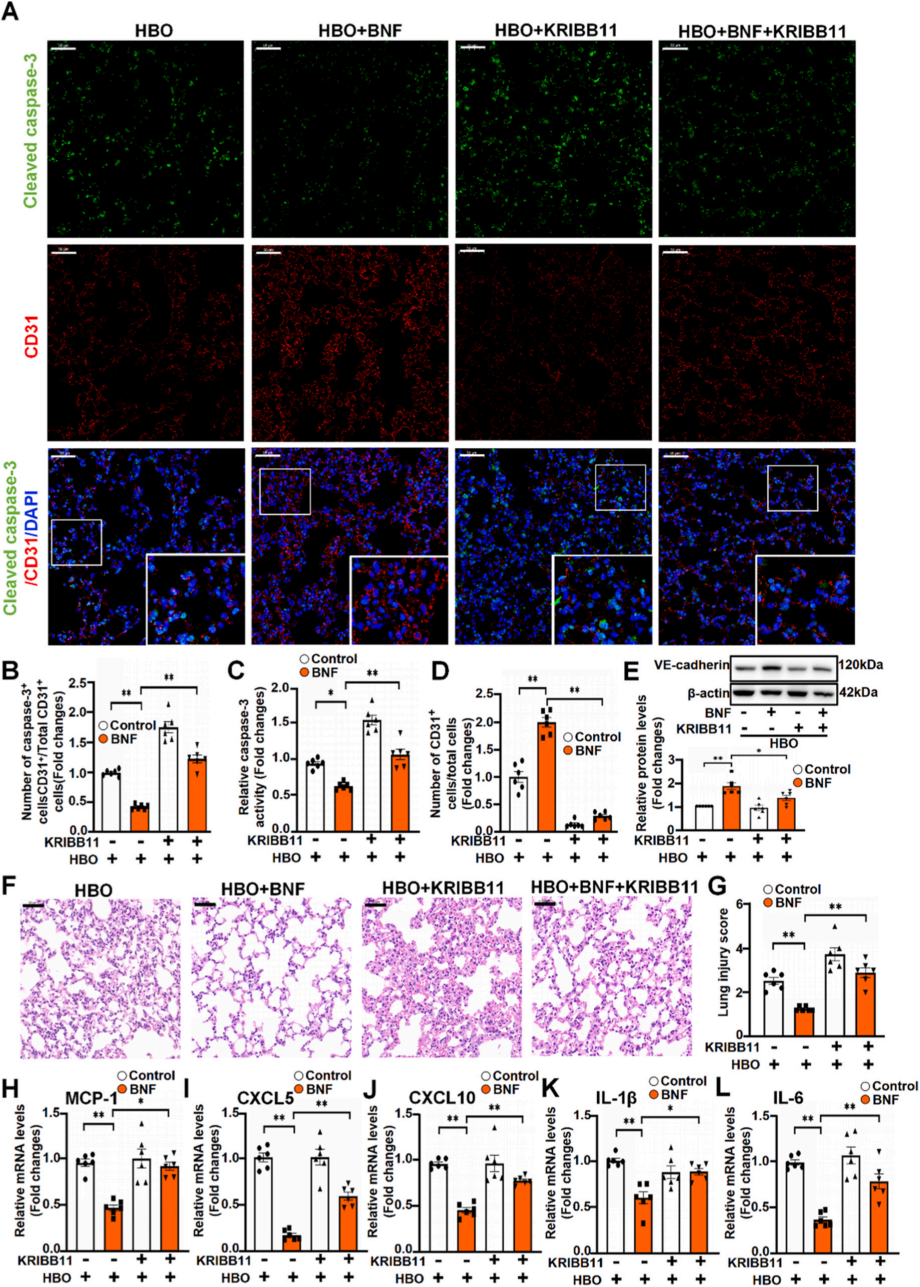
HSF1 inhibitor abolishes the protective effects of CYP1A inducer against HBO-induced pulmonary endothelial apoptosis and lung **injury. A,** Immunofluorescence staining of Cleaved caspase-3 (green) expression levels in the lung tissue of mice exposed to HBO after BNF and KRIBB11 treatment. The cell nuclei were stained with DAPI. Scale bar = 50 μm. **B,** The number of cells with Cleaved caspase-3 (green) and CD31 overlap is presented in a histogram (n = 6). **C,** Detection of caspase-3 activity (n = 6). **D,** Immunofluorescence staining of CD31 (red) in the lung tissue of HBO-exposed mice treated with CYP1A inducer BNF and HSF1 inhibitor KRIBB11. The number of cells with CD31 and DAPI overlap is presented in a histogram (n = 6). **E,** Western Blot detection of protein expression levels of VE-cadherin in the lung tissue of HBO-exposed mice treated with BNF and KRIBB11 (n = 6). The corresponding histograms are shown below the bands. **F,** Staining of lung tissue with hematoxylin and eosin to show the pathological changes of BNF and KRIBB11-treated HBO-exposed mouse lungs. Original magnification, ×400. Scale bar corresponds to 50 μm. **G,** Histogram of lung tissue pathological injury score (n = 6). **H-L,** Detection of mRNA expression levels of inflammatory chemokines MCP-1, CXCL10, IL-6, IL-1β, and CXCL5 in the lung tissue of HBO-exposed mice treated with BNF and KRIBB11 by qRT-PCR (n = 6). Data are presented as mean ± SEM. ∗p < 0.05, ∗∗p < 0.01.

Collectively, these results indicate that HSF1 inhibitor decreases SDHC expression and abolishes the protective effects of CYP1A inducer against HBO-induced mitochondrial oxidative stress, mitochondrial dysfunction and endothelial cell apoptosis in lung tissues.

### 3.5 CYP1A deficiency decreases SDHC expression and aggravates HBO-induced mitochondrial oxidative stress, mitochondrial dysfunction and endothelial cell apoptosis in lung tissues

As described above, CYP1A deficiency significantly aggravated HBO-induced endothelial injury and acute lung injury. It was found that CYP1A deficiency also reduced SDHC expression and mitochondrial complex II activity in lung tissues of HBO-exposed mice (Fig. 9A–C). We next examined the effect of CYP1A deficiency on mitochondrial oxidative stress, mitochondrial dysfunction and endothelial cell apoptosis in lung tissues of HBO-exposed mice. As shown in Fig. 9D–H and Supplemental Fig. S13A, CYP1A deficiency significantly increased 8-OHdG-positive cells, mitochondrial superoxide production, as well as decreases in mitochondrial membrane potential and ATP production in lung tissues of HBO-exposed mice. Moreover, CYP1A deficiency caused significant increases in caspase-3 activity as well as the percentage of cleaved caspase-3/CD31^+^ cells in total CD31^+^ cells in lung tissues of HBO-exposed mice (Fig. 9I–K and Supplemental Fig. S13B). These results indicate that CYP1A deficiency decreases SDHC expression and aggravates HBO-induced mitochondrial oxidative stress, mitochondrial dysfunction and endothelial cell apoptosis in lung tissues.

**Fig. 9:**
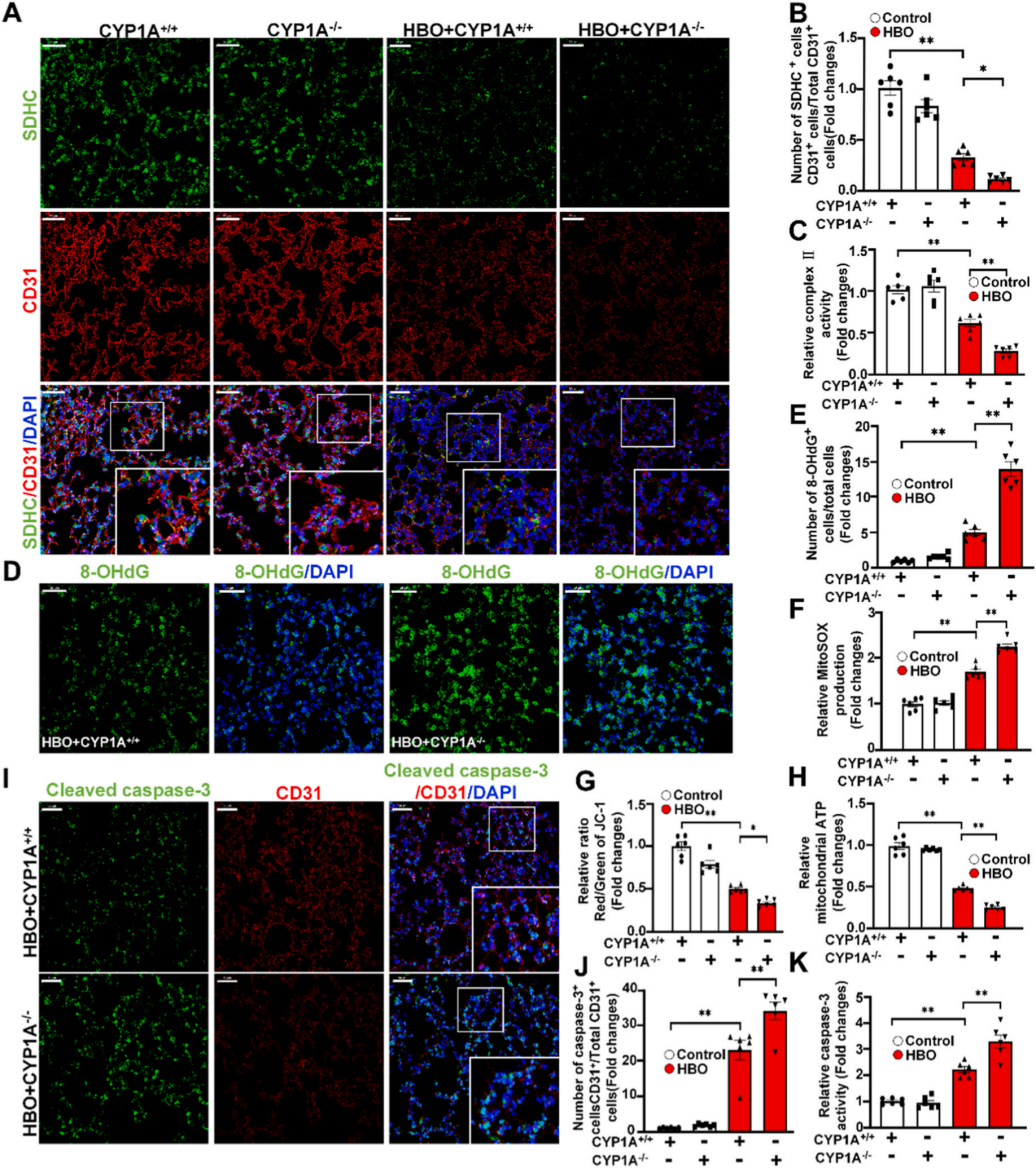
Knockout of the CYP1A gene downregulates the expression of SDHC, exacerbating mitochondrial oxidative stress, mitochondrial dysfunction, and early cell apoptosis in the lung tissue of mice exposed to HBO. A, Immunofluorescence staining of the expression levels of SDHC (green) and endothelial cell marker CD31 (red) in the lung tissue of CYP1A knockout mice after HBO exposure. The cell nuclei were stained with DAPI. Scale bar = 50 μm. **B,** The proportion of cells co-positive for SDHC and CD31 among all CD31-positive cells is shown in a histogram (n = 6). **C,** After CYP1A knockout, the activity of mitochondrial complex II in the lung tissue of HBO-exposed mice was detected (n = 6). **D,** Immunofluorescence staining of the expression level of 8-OHdG (green) in the lung tissue of CYP1A knockout mice after HBO exposure. The cell nuclei were stained with DAPI. Scale bar = 50 μm. **E,** The number of cells with overlapping 8-OHdG (green) and DAPI was shown in a histogram (n = 6). **F,** After CYP1A knockout, the fluorescence intensity of MitoSOX in the mitochondria of lung tissue was detected (n = 6). **G,** After CYP1A knockout, the ratio of fluorescence intensity of JC-1 aggregates (red) to monomers (green) in the mitochondria of lung tissue was detected (n = 6). **H,** After CYP1A knockout, the ATP content in the mitochondria of lung tissue was detected (n = 6). **I,** Immunofluorescence staining of the expression level of Cleaved caspase-3 (green) in the lung tissue of CYP1A knockout mice after HBO exposure. The cell nuclei were stained with DAPI. Scale bar = 50 μm. **J,** The number of cells with overlapping Cleaved caspase-3 (green) and CD31 was shown in a histogram (n = 6). **K,** Detection of caspase-3 activity (n = 6). Data are presented as the mean ± SEM. ∗p < 0.05, ∗∗p < 0.01.

## 4 Discussion

The CYP enzyme superfamily comprises membrane-bound heme-containing monooxygenases, among which the CYP1 family plays a key role in catalyzing oxidation and reduction reactions in vivo. Notably, CYP1A enzymes exhibit antioxidant properties and may confer protection by mitigating oxidative stress [17,36]. Altered expression of pulmonary CYP1 family members has been observed across various lung pathologies. For instance, CYP1A1 expression is significantly upregulated in mouse models of LPS-induced sepsis, TDI-induced asthma, hyperoxic lung injury, and following intratracheal instillation of diesel exhaust particles (DEP) [[37], [38], [39], [40], [41], [42]]. Similarly, elevated CYP1A1 levels have been detected in tracheal aspirates from ARDS patients compared to ventilated controls [43], and in human lung epithelial cell lines—including A549, BEAS-2B, and H441, within 24 h of hyperoxia exposure [44]. By analyzing the public available single-cell transcriptomic data, we found that CYP1A1 and CYP1A2 are predominantly expressed in alveolar epithelial and pulmonary microvascular endothelial cells in the lung. Given that hyperbaric oxygen (HBO) exposure has been shown to impair both epithelial and endothelial integrity [21], yet no prior studies have investigated CYP1 family dynamics specifically in lung endothelial cells under pathological conditions, this study demonstrates for the first time that CYP1A1 and CYP1A2, but not CYP1B1, are upregulated in lung tissues and microvascular endothelial cells following HBO exposure, concomitant with endothelial barrier dysfunction. These findings suggest a critical involvement of CYP1A isoforms in HBO-induced pulmonary endothelial injury.

Previous studies have demonstrated that CYP1A exerts protective effects against lung injury induced by various stressors, including LPS-induced sepsis and hyperoxic or hypoxic environments, primarily through investigations utilizing genetically engineered CYP1A-deficient mouse models or pharmacological CYP1A inducers. For instance, CYP1A1 deficiency has been shown to exacerbate LPS-induced acute lung injury, accompanied by elevated levels of pro-inflammatory mediators such as TNF-α, IL-1β, IL-6, and nitric oxide in lung tissue [37]. Furthermore, Shertzer and Lingappan et al. reported that CYP1A2-knockout mice exhibit increased susceptibility to hyperoxia-induced lung injury, with enhanced generation of reactive oxygen species [45,46]. Pharmacological induction of CYP1A1 and CYP1A2 using omeprazole or BNF has been found to attenuate hyperoxia-induced pulmonary edema, alveolar hemorrhage, and leukocyte infiltration, thereby mitigating lung injury [25,47,48]. These findings collectively suggest a potential role for CYP1A in preserving pulmonary capillary barrier integrity. In the present study, by employing both CYP1A1/CYP1A2 double-knockout and BNF-treated murine models, we provide compelling evidence that CYP1A deficiency aggravates HBO-induced pulmonary endothelial permeability, histopathological damage, and inflammatory responses, whereas pharmacological induction of CYP1A ameliorates these adverse effects. Our results suggest that the upregulation of CYP1A may constitute a novel adaptive protective mechanism against HBO-induced pulmonary endothelial barrier dysfunction. Notably, in contrast to these findings and previous reports, Ha AW et al. recently demonstrated that methicillin-resistant *Staphylococcus aureus* (MRSA) promotes CYP1A1 expression, and that suppression of CYP1A1 via siRNA knockdown or inhibition with rhapontigenin attenuates MRSA-induced endothelial cell permeability and inflammation [49]. This discrepancy highlights the context-dependent role of the CYP1A family in modulating pulmonary endothelial barrier function, which appears to vary according to the underlying etiology of acute lung injury.

Notably, we found that BNF conferred modest yet statistically significant protection in CYP1A-deficient mice, in contrast to the robust protection observed in wild-type littermates, indicating that while CYP1A is the principal mediator of BNF's protective action, residual protection persists in its absence. BNF is a well-characterized aryl hydrocarbon receptor (AhR) ligand, and AhR is a pleiotropic transcription factor known to regulate not only CYP1A but also multiple endothelial-protective genes including Nrf2 and NQO1 [50], particularly under oxidative or inflammatory stress. Thus, we propose that BNF may engage CYP1A-independent transcriptional programs that contribute to endothelial stabilization, a hypothesis that merits mechanistic investigation in future studies.

Through RNA-Seq analysis of lung tissues from CYP1A-deficient or CYP1A inducer-treated mice exposed to hyperbaric oxygen, we identified CYP1A as a potential upstream regulator of HSF1. Both in vivo and in vitro evidence demonstrated that activation of the HSF1 signaling pathway is critically involved in the protective effects of CYP1A against hyperbaric oxygen-induced pulmonary endothelial barrier dysfunction. This finding aligns with previous studies reporting the protective role of HSF1 in lung injury caused by LPS [[51], [52], [53]] and sepsis [54]. As the master transcriptional regulator of the heat shock response (HSR) in mammalian cells, HSF1 maintains protein homeostasis by promoting the refolding or degradation of misfolded proteins that accumulate under cellular stress [[55], [56], [57], [58], [59], [60], [61]]. However, prior investigations into the lung-protective mechanisms of HSF1 have primarily emphasized its antioxidant and anti-inflammatory properties, leaving its detailed molecular pathways incompletely understood. In this study, we show that both CYP1A inducer and HSF1 agonist alleviate hyperbaric oxygen-induced apoptosis and increased permeability in pulmonary microvascular endothelial cells by suppressing mitochondrial oxidative stress and restoring mitochondrial function. Notably, the protective effects of CYP1A inducers against hyperbaric oxygen-induced mitochondrial oxidative stress, mitochondrial dysfunction, and endothelial cell apoptosis are abrogated by HSF1 inhibition. These results indicate that CYP1A upregulation ameliorates hyperbaric oxygen-induced endothelial injury through direct activation of the HSF1-dependent mitochondrial protective pathway.

In fact, accumulating studies have demonstrated that HSF1 activation reduces cell apoptosis by suppressing oxidative stress and improving mitochondrial function [32,33,62,63]; however, the underlying molecular mechanisms remain less well understood. In a recent study, Dong et al. first identified succinate dehydrogenase C (SDHC) as a direct transcriptional target of HSF1. Deletion of HSF1 dysregulates multiple genes associated with leukemic stem cell stemness and suppresses mitochondrial oxidative phosphorylation through downregulation of SDHC, a defect that can be largely rescued by forced expression of SDHC [35]. In the present study, we identified two conserved consensus HSF1-binding motifs within the proximal region of both the mouse and human SDHC promoters. HSF1 inhibitor significantly suppressed the BNF-induced SDHC upregulation in HBO-exposed pulmonary endothelial cells. Moreover, ChIP-qPCR assays confirmed that BNF treatment markedly enhanced HSF1 occupancy at both consensus binding sites on the SDHC promoter, a response that was largely suppressed by HSF1 inhibitor. Collectively, these findings provide direct mechanistic evidence that CYP1A-mediated HSF1 activation drives SDHC transcription.

SDHC serves as a critical anchor subunit of succinate dehydrogenase (SDH), also known as respiratory complex II, in the mitochondrial inner membrane [64,65]. Previous studies have shown that inactivation of SDHC leads to reduced mitochondrial oxygen consumption and excessive ROS production, thereby triggering mitochondrial oxidative stress and dysfunction, ultimately inducing apoptosis and cellular toxicity in skeletal muscle cells [66], hematopoietic stem cells [67], fibroblasts [68,69], keratinocytes [70], and hepatocytes [71]. Conversely, SDHC overexpression has been shown to enhance mitochondrial respiration, alleviate oxidative phosphorylation impairment, and reduce cell damage in skeletal muscle cells and tubular epithelial cells [72,73]. To date, no studies have investigated the role of SDHC in pulmonary parenchymal cells. In this study, we report for the first time that CYP1A inducer upregulates SDHC expression in a HSF1-dependent manner. Furthermore, the protective effects of CYP1A inducer and HSF1 agonist against HBO-induced mitochondrial oxidative damage and pulmonary vascular endothelial cell injury were abolished by SDHC knockdown. These findings suggest that the endothelial-protective effects of CYP1A during HBO-induced acute lung injury are at least partially mediated by activation of the HSF1/SDHC signaling pathway.

Preclinical and clinical studies have identified several CYP1A inducers, each with unique pros and cons. Omeprazole, a common proton-pump inhibitor, can induce CYP1A1 and CYP1A2 in human hepatocytes and alimentary tissues via AhR-dependent transcriptional activation [[74], [75], [76]]. However, it activates AhR through a non - genomic, tyrosine - kinase - mediated pathway [77,78], leading to less predictable induction compared to classic AhR ligands like BNF or 3-methylcholanthrene. Also, there are significant species differences: it is potent in humans but has little effect on CYP1A in rats, mice, and rabbits [79], making preclinical to human translation difficult. BNF, a non-carcinogenic synthetic flavonoid and classic AhR agonist, has been used in experiments to induce CYP1A and protect against toxic insults like aristolochic acid-induced nephrotoxicity [80]. But it is only a research tool for CYP1A induction in preclinical and mutagenicity assays [81], and its efficacy in lung injury, oral bioavailability, and human safety are largely unknown. Timing of administration is a major limitation. CYP1A1 and CYP1A2 are AhR-regulated genes, and their protective effect depends on new enzyme synthesis, which takes time. This is a big challenge in acute HBO therapy.

Drug-drug interactions represent another significant concern, as CYP1A enzymes metabolize clinically important drugs, including theophylline, caffeine, clozapine, and olanzapine [82]. CYP1A induction may accelerate the systemic clearance of co-administered medications, potentially resulting in subtherapeutic drug concentrations and therapeutic failure. Moreover, CYP1A exhibits a well-documented functional duality: while CYP1A1 and CYP1A2 mitigate ROS-derived lipid peroxidation products in HBO-induced ALI, they also catalyze the bioactivation of procarcinogens (e.g., polycyclic aromatic hydrocarbons and heterocyclic aromatic amines) into reactive electrophilic intermediates that form DNA adducts [83,84]. Although sustained CYP1A induction could theoretically elevate cancer risk in individuals with chronic environmental carcinogen exposure, this risk remains hypothetical, strictly dependent on concurrent exposure to relevant procarcinogenic substrates, and has not been directly observed or validated in clinical studies. Collectively, although pharmacological CYP1A induction holds promise as a preventive strategy against HBO-induced ALI, its clinical translation necessitates rigorous evaluation of inducer selection, tissue-specific efficacy, timing of administration, potential drug-drug interactions, and long-term safety implications. Future studies should prioritize the development of lung-targeted CYP1A inducers with favorable safety profiles and the validation of predictive biomarkers to identify HBO patients at highest risk for ALI who would derive the greatest benefit from prophylactic CYP1A induction.

In this study, we utilized a male mouse model to investigate HBO-induced pulmonary endothelial barrier dysfunction and acute lung injury. We acknowledge that a major limitation of our current work is the lack of parallel investigations in female mice. Previous studies have demonstrated sex-specific differences in susceptibility to hyperoxic lung injury, with female mice generally exhibiting greater tolerance than male mice. This sexual dimorphism is attributable, at least in part, to differential expression and regulation of CYP1A enzymes between sexes [46,85,86]. Future studies are warranted to further investigate the protective effects of CYP1A enzymes in females under hyperbaric oxygen conditions, which is essential for fully elucidating the generalizability of this protective mechanism.

In summary, this study demonstrates for the first time that upregulation of CYP1A may represent a novel adaptive protective mechanism against hyperbaric oxygen-induced pulmonary endothelial barrier dysfunction and acute lung injury. Mechanistically, CYP1A upregulation alleviates hyperbaric oxygen-induced endothelial injury by suppressing mitochondrial oxidative stress and restoring mitochondrial function through activation of the HSF1/SDHC signaling pathway. Therefore, targeting the CYP1A/HSF1/SDHC pathway via genetic or pharmacological interventions to mitigate mitochondrial dysfunction may provide promising therapeutic strategies for hyperbaric oxygen-induced acute lung injury.

**Table:** Abbreviations

HBO	Hyperbaric oxygen
ALI	Acute lung injury
CYP	Cytochrome P450
HSF1	Heat shock factor 1
SDHC	Succinate dehydrogenase C
ATA	Atmospheres absolute
MLVEC	Mouse lung vascular endothelial cell
BNF	β-naphthoflavone
FITC	Fluorescein isothiocyanate carboxymethyl
VE-cadherin	Vascular endothelial cadherin
CD31	Platelet endothelial cell adhesion molecule-1
GSEA	Gene Set Enrichment Analysis
DEGs	Differential expressed genes
GO	Gene Ontology
LPS	Lipopolysaccharides

## Ethics approval and consent to participate

This study was conducted in compliance with the National Institutes of Health Guide for the Care and Use of Laboratory Animals. The protocol was approved by the Ethics Committee of Naval Medical Center (Ethics number: NMC-2021021). This study does not involve human subjects, human material, or human data.

## Data availability

Data will be made available on request.

## Funding

The study was supported by 10.13039/501100001809National Natural Science Foundation of China (No. 32171124, 32271180, 32471185, 31971101, and 81971780), a departmental fund (NO. 21AH0102), Yi Zhang Talent Cultivation Program of Naval Medical University, (NO. JCYZRC-D-025). Fund of the Basic Medical College of Naval Medical University, (No. JCXYXZ-001).

## CRediT authorship contribution statement

**Jing-Qian Huang:** Data curation, Formal analysis, Software, Validation, Visualization, Writing – original draft, Writing – review & editing. **Peng-Xiang Wang:** Data curation, Formal analysis, Validation, Visualization, Writing – review & editing. **Xiao-Yong Zhang:** Data curation, Investigation, Writing – review & editing. **Yi-Ting You:** Data curation, Validation. **Jun-Hui Zhan:** Formal analysis, Software. **Dan-Hong Xu:** Formal analysis, Software. **Yu-Jian Liu:** Investigation, Resources. **Xiao-Yan Zhu:** Conceptualization, Funding acquisition, Methodology, Project administration, Resources, Supervision, Validation, Writing – review & editing. **Xiao-Chen Bao:** Conceptualization, Formal analysis, Methodology, Supervision, Validation, Writing – review & editing.

## Declaration of competing interest

I have nothing to declare.

## Figures and Tables

**figs1:**
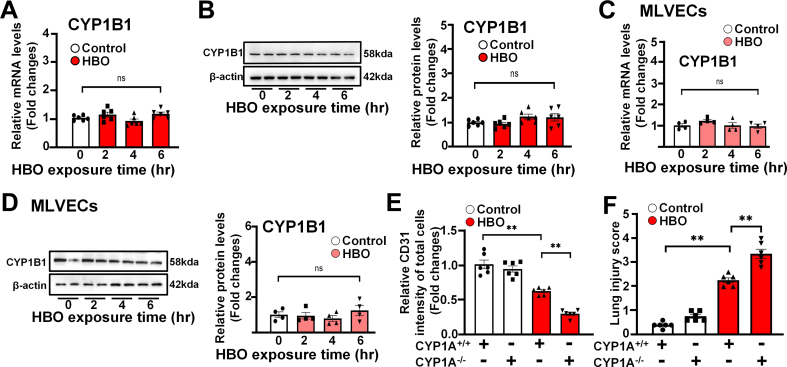


**figs2:**
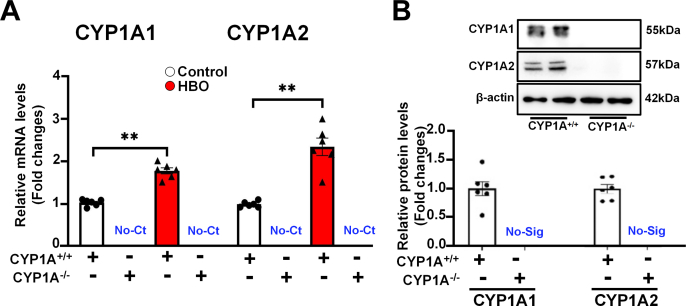


**figs3:**
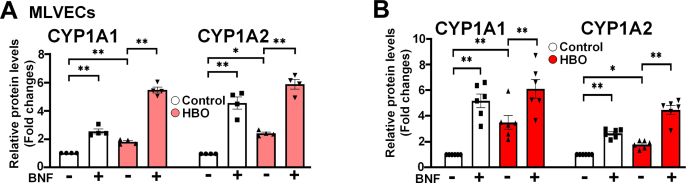


**figs4:**
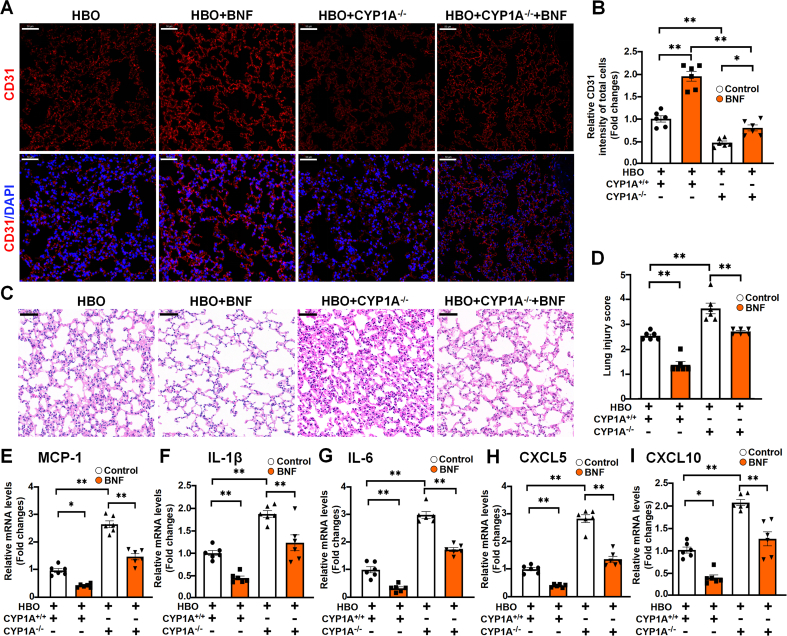


**figs5:**
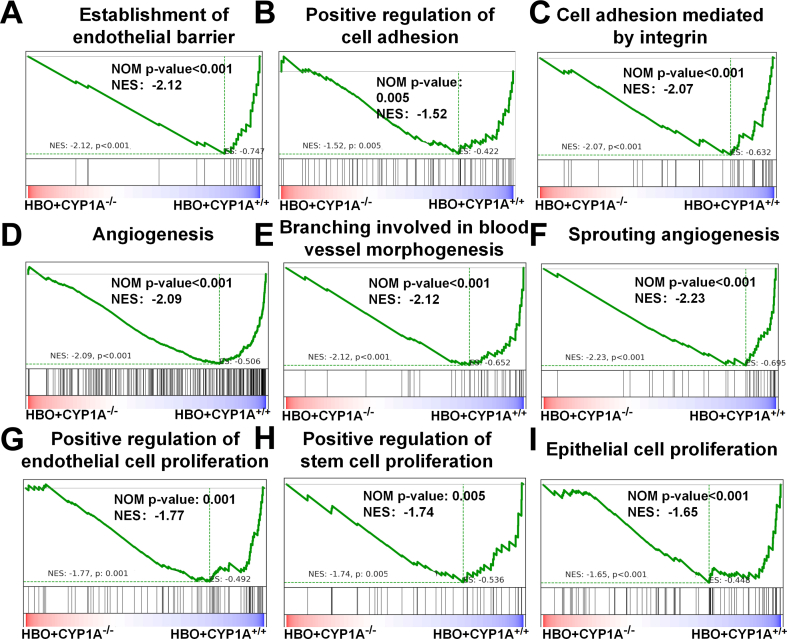


**figs6:**
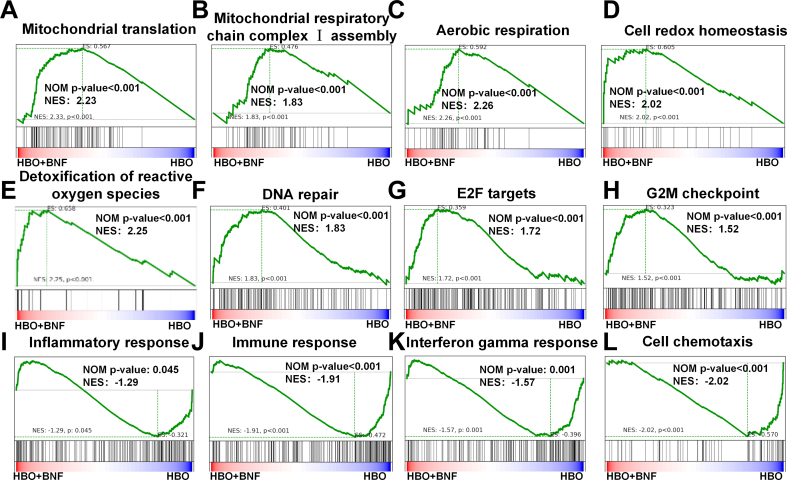


**figs7:**
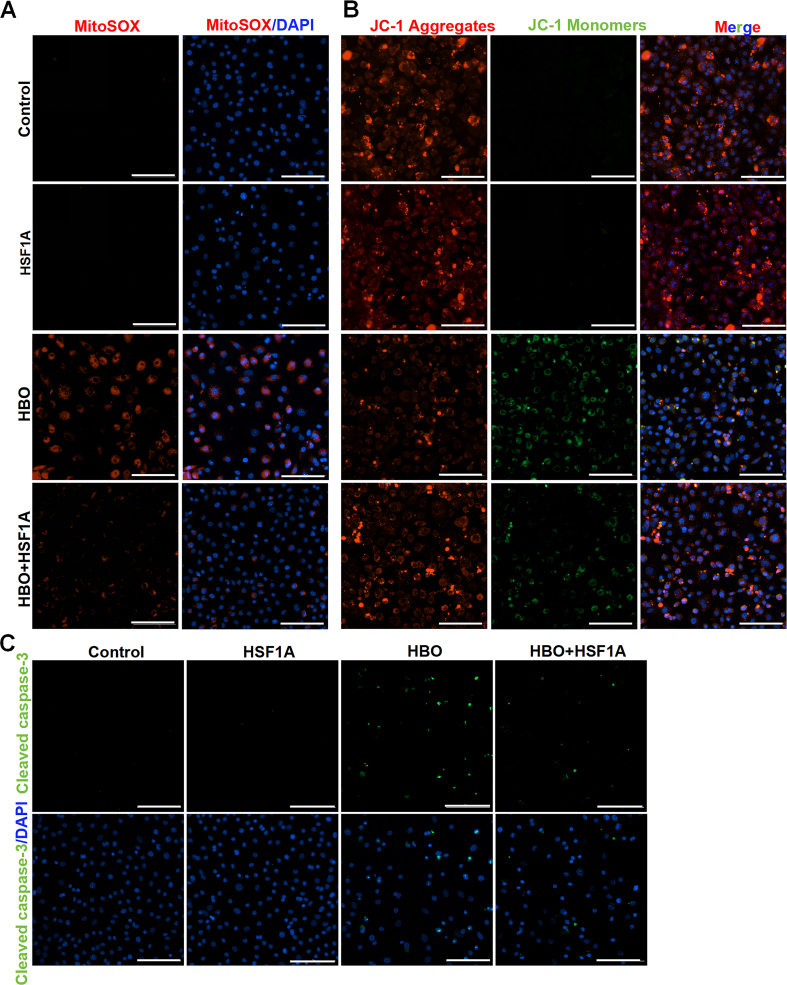


**figs8:**
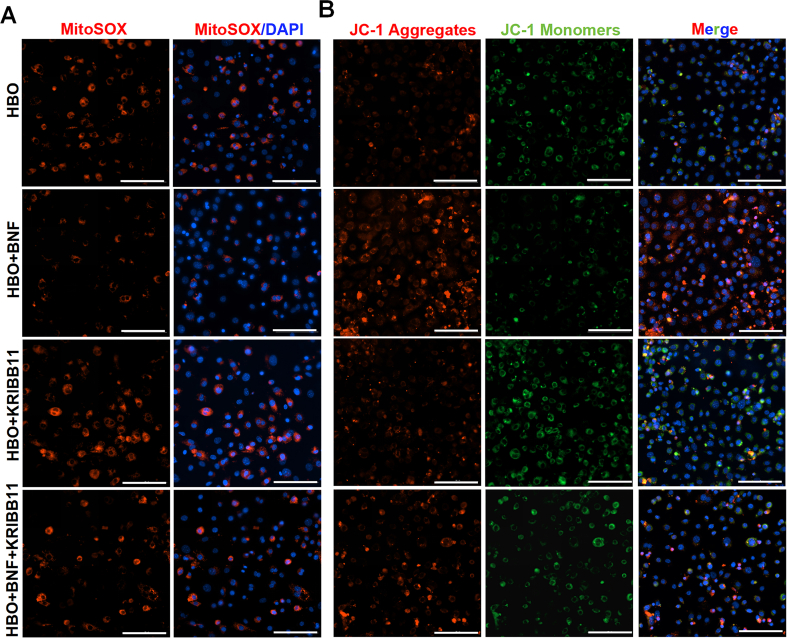


**figs9:**
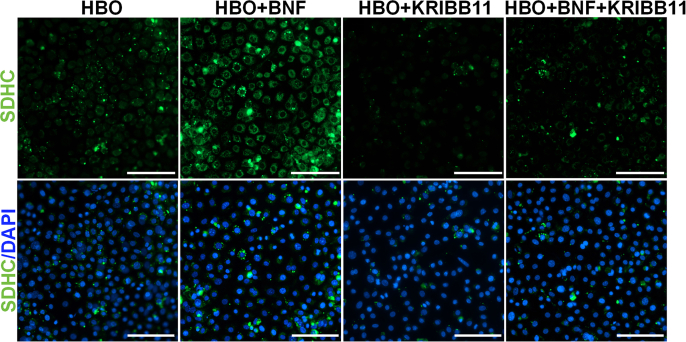


**figs10:**
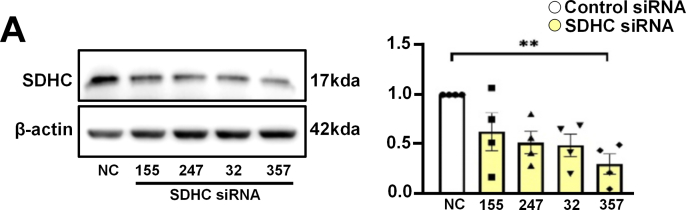


**figs11:**
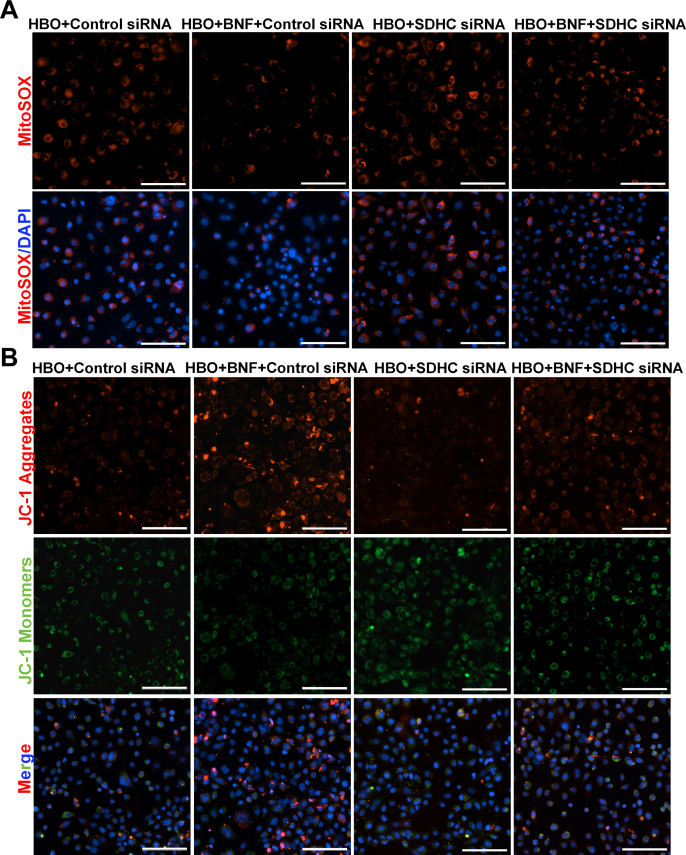


**figs12:**
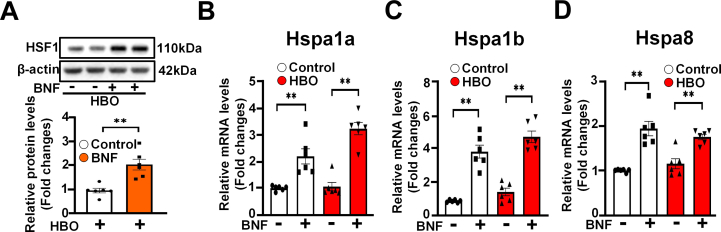


**figs13:**