# Supplementary material for: The CYP1A-HSF1 axis alleviates mitochondrial oxidative stress to limit HBO-induced lung endothelial barrier damage
Source: Redox Biol. 2026 Jul 16;96:104308. doi: 10.1016/j.redox.2026.104308 (PMC13452367; doi:10.1016/j.redox.2026.104308)
Supplement: Supplementary file 1 [file mmc1.docx]

**Supplementary Table 1**

| **Primer sequences used in the real-time quantitative PCR** | | |
| --- | --- | --- |
| Gene names | Forward (5'to3') | Reverse (5'to3') |
| Mouse β-actin | CTGTATGCCTCTGGTCGTAC | TGATGTCACGCACGATTTCC |
| Mouse Il-1β | TGACGTTCCCATTAGACAACTG | CCGTCTTTCATTACACAGGACA |
| Mouse Il-6 | GAACAACGATGATGCACTTGC | CTTCATGTACTCCAGGTAGCTATGGT |
| Mouse Mcp-1 | GCATCCACGTGTTGGCTCA | CTCCAGCCTACTCATTGGGATCA |
| Mouse Cxcl5 | TGATCGCTAATTTGGAGGTGAT | TAGCTTTCTTTTTGTCACTGCC |
| Mouse Cxcl10 | CAACTGCATCCATATCGATGAC | GATTCCGGATTCAGACATCTCT |
| Mouse Hspa1a | TTGTGTATTGCACGTGGGCT | TGGTGCTGTCACTTCACCTC |
| Mouse Hspa1b | GCTGATCCAGGTGTACGAGG | GGTGCCCAAGCAGCTATCAA |
| Mouse Hsph1 | GAGGCGCCGGTGAGTAAAAT | GAGCCTACGTCTAGCCCAAC |
| Mouse Hspa8 | CGTACCTCGGAAAGGCAACA | TCAAAAGTGCCACCTCCCAA |
| Mouse Bag3 | GAAGCCGAAACACCCCCTAA | CTGAACCTTCCTGACGCCAT |
| Mouse Sdhc | TCTTCCCGCTCATGTACCAC | GACAACACAGCAAGAACCACGA |
| Mouse Cyp1a1 | AGGTGGTAGTTCTTGGAGCTT | GCAGAATACGGTGACAGCCA |
| Mouse Cyp1a2 | GCTTCTCCATAGCCTCGGAC | CTGGCTGACTGGTTCGAAGT |
| Mouse Cyp1b1 | ATCGGATGGCACACCACG | ACTATCGCACTCCCCTTCCT |
|  |  |  |
| **Primer sequences used in the Chip-qPCR** | | |
| SDHC promoter | Forward (5'to3') | Reverse (5'to3') |
| SDHC promoter region (-1416/-1408) | GAGAGGGTTGGAGCGATGTC | CACTCTCCAGAGACAGGTAAA |
| SDHC promoter region (-171/-161) | CGCTAGCTAGAAACTGGCGCGTGA | GTTCCGGGCTTGGTGGATGGTACT |
